# IL‐36*γ* and IL‐36Ra Reciprocally Regulate Colon Inflammation and Tumorigenesis by Modulating the Cell–Matrix Adhesion Network and Wnt Signaling

**DOI:** 10.1002/advs.202103035

**Published:** 2022-02-04

**Authors:** Wei Yang, Hong‐Peng Dong, Peng Wang, Zhi‐Gao Xu, Jiahuan Xian, Jiachen Chen, Hai Wu, Yang Lou, Dandan Lin, Bo Zhong

**Affiliations:** ^1^ Department of Gastrointestinal Surgery Medical Research Institute Zhongnan Hospital of Wuhan University Wuhan 430071 China; ^2^ Department of Pulmonary and Critical Care Medicine Zhongnan Hospital of Wuhan University Wuhan 430071 China; ^3^ Frontier Science Center for Immunology and Metabolism Wuhan University Wuhan 430071 China; ^4^ Department of Virology College of Life Sciences Wuhan University Wuhan 430072 China; ^5^ Institute of Hepatobiliary Diseases and Transplant Center Zhongnan Hospital of Wuhan University Wuhan 430071 China; ^6^ Yurogen Biosystems LLC (Wuhan) 666 Gaoxin Avenue, Building C6, Donghu District Wuhan 430064 China; ^7^ Cancer Center Renmin Hospital of Wuhan University Wuhan 430061 China; ^8^ Wuhan Research Center for Infectious Diseases and Cancer Chinese Academy of Medical Sciences Wuhan 430071 China

**Keywords:** cell–matrix adhesion, colon cancer, IL‐36*γ*, IL‐36Ra, tumor microenvironment, Wnt signaling

## Abstract

Inflammatory bowel disease and colorectal cancer are associated with dysregulation of cytokine networks. However, it is challenging to target cytokines for effective intervention because of the overlapping functions and unpredictable interactions of cytokines in such diverse networks. Here, it is shown that IL‐36*γ* and IL‐36Ra, an agonist and an antagonist for IL‐36R signaling respectively, reciprocally regulate the experimental colitis and the colon cancer development in mice. Knockout or neutralization of IL‐36*γ* alleviates dextran sulfate sodium (DSS)‐induced colitis and inhibits colon cancer development, whereas knockout of IL‐36Ra exacerbates DSS‐induced colitis and promotes colonic tumorigenesis in multiple colon cancer models in mice. Mechanistically, IL‐36*γ* upregulates extracellular matrix and cell–matrix adhesion molecules and facilitates Wnt signaling, which is mitigated by IL‐36Ra or IL‐36*γ* neutralizing antibody. Consistently, IL‐36*γ* levels are positively correlated with extracellular matrix levels and *β*‐catenin levels in human colorectal tumor biopsies. These findings suggest the critical role of IL‐36*γ* and IL‐36Ra in gut inflammation and tumorigenesis and indicate that targeting the IL‐36*γ*/IL‐36Ra signal balance provides potential therapeutic strategy for inflammatory bowel disease and gastrointestinal cancers.

## Graphical abstract

Inflammatory bowel disease and colorectal cancer are associated with dysregulation of cytokine networks. Here, it is shown that IL‐36*γ* promotes and IL‐36Ra reciprocally inhibits the experimental colitis and the colon cancer development in mice by modulating the cell–matrix network and Wnt signaling. These findings indicate that targeting IL‐36*γ* provides potential therapeutic strategy for inflammatory bowel disease and gastrointestinal cancers.

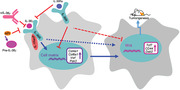

## 1 Introduction

Aberrant cytokine expression and signaling are closely associated with inflammatory bowel disease (IBD) and colorectal cancer (CRC) that cause serious health problems worldwide.^[^ ^1^, ^2^ ^]^ It has been proposed that the cytokine milieu supporting unspecific, chronic and protumorigenic inflammation in the microenvironment critically determines disease progression of IBD and CRC.^[^ ^3^ ^]^ In support of this notion, genome‐wide association studies (GWAS) and next‐generation sequencing analyses (NSG) have identified a number of mutations or single nucleotide polymorphisms (SNPs) in the cytokine signaling pathways associated with IBD and CRC.^[^ ^4^, ^5^, ^6^ ^]^ Accordingly, therapeutic strategies targeting the proinflammatory cytokines have been developed for treatment of IBD and CRC. For example, antitumor necrosis factor agents have been clinically approved to treat IBD with limitations such as loss of response in about two thirds of patients and increased risk of infections.^[^ ^7^ ^]^ However, completed clinical trials have shown either limited but significant clinical remission with a follow‐up of 6–22 weeks (such as anti‐IL‐12p40 or anti‐IL‐23p19) or no improvement (such as antibodies against IL‐17A, IL‐1, or IL‐6) for IBD.^[^ ^7^ ^]^ In addition, clinical trials with blockades against proinflammatory cytokines in CRC have so far shown little to no effect in disease control.^[^ ^3^, ^8^, ^9^, ^10^ ^]^ Although the reasons behind these phenomena are unclear, the available data indicate challenges to modulate cytokine networks for treatment of IBD and CRC.

The extracellular matrix (ECM) is a complex and highly dynamic structure that plays critical roles during homeostasis and disease pathophysiology in the gastrointestinal tract.^[^ ^11^, ^12^ ^]^ The ECM directly provides biochemical and mechanical signals for cells and indirectly regulates extracellular stimuli‐triggered signaling in cells, thereby controlling the fate of gastrointestinal epithelial cells such as proliferation, apoptosis, or malignancy.^[^ ^11^ ^]^ In addition, the ECM components also interact with cell–matrix adhesion molecules to regulate the permeability of the gut epithelium and the migration of epithelial cells.^[^ ^13^, ^14^ ^]^ Concurrently, alterations in the cellular signaling under inflammatory or under tumor promoting conditions can change the composition and structure of ECM and cell–matrix adhesion molecules.^[^ ^12^ ^]^ Consistently, dysregulated expression of ECM and cell–matrix adhesion molecules is observed with IBD and CRC and predicts prognosis. For example, collagens are a major type of gut ECM components and the types and contents of collagens have altered significantly in the gut mucosa and the serum of patients with IBD and CRC and are associated with the severity of disease.^[^ ^15^, ^16^, ^17^, ^18^, ^19^, ^20^ ^]^ Therefore, understanding the dynamic bidirectional crosstalk between resident cells and the ECM and identifying key mediators between the crosstalk are central to elucidating the mechanisms of IBD and CRC and subsequent developing medicines for the related diseases.

IL‐36 belongs to the IL‐1 family of cytokines, including IL‐36*α*, IL‐36*β*, and IL‐36*γ* that bind to IL‐36 receptor (IL‐36R) and IL‐1 receptor accessory protein (IL‐1RacP) to initiate signaling.^[^ ^21^, ^22^, ^23^ ^]^ IL‐36R signals through myeloid differentiated protein 88 (MyD88) to activate mitogen‐activated protein kinase (MAPK) and NF‐*κ*B, and thereby promotes inflammation by inducing expression of an array of proinflammatory cytokines.^[^ ^22^ ^]^ IL‐36Ra is a natural antagonist of IL‐36R and binds IL‐36R with higher affinity than the IL‐36 agonistic cytokines.^[^ ^24^ ^]^ Increased levels of IL‐36 cytokines have been found in the skin lesions of patients with plaque psoriasis and overexpression of IL‐36*α* in mouse keratinocytes results in psoriatic skin abnormalities that are aggravated by *Il1f5* (encoding IL‐36Ra) deficiency.^[^ ^25^ ^]^ In addition, humans carrying mutations in *IL1F5* develop generalized pustular psoriasis (GPP),^[^ ^26^, ^27^ ^]^ suggesting crucial roles of imbalanced IL‐36R signaling in promoting skin inflammation. It has been observed that IL‐36*α* and IL‐36*γ* are upregulated in the colon mucosa of patients with IBD.^[^ ^28^, ^29^, ^30^ ^]^ However, the roles of IL‐36 cytokines in colon inflammation and tumorigenesis remain uninvestigated.

In this study, we have discovered that deficiency in IL‐36*γ* and IL‐36Ra results in hypo‐ and hyper‐sensitivity to DSS‐induced colitis and colon cancer induction with multiple models, respectively. Mechanistically, IL‐36*γ* upregulates expression of an array of genes encoding ECM and cell–matrix adhesion molecules and facilitates Wnt signaling, which is mitigated by IL‐36*γ* neutralizing antibody or IL‐36Ra. These findings collectively suggest critical roles of IL‐36*γ* and IL‐36Ra in colitis and CRC, which serve as potential therapeutic targets.

## 2 Results

### 2.1 The *Il1f9* ^−/−^ Mice Exhibit Hyposensitivity to DSS‐Induced Colitis

Our RNA sequencing data suggest that of the three IL‐36 cytokines, IL‐36*γ* (encoded by *Il1f9*) is the most abundantly expressed in the colon tissues of DSS‐treated mice.^[^ ^31^ ^]^ Importantly, *IL1F9* is significantly upregulated in the inflamed colon mucosa of IBD patients compared to the normal colon tissues.^[^ ^28^, ^29^, ^30^ ^]^ We further found that the numbers of Paneth cells (lysozyme^+^) were significantly increased in the crypts of small intestines of *Il1f9* ^−/−^ mice compared to *Il1f9* ^+/+^ mice, whereas the proliferative cells (Ki67^+^) in the crypts were slightly decreased in the small intestines and colons of *Il1f9* ^−/−^ mice compared to *Il1f9* ^+/+^ mice (Figure S1A, Supporting Information). Consistently, we found that IL‐36*γ* promotes the proliferation of the HCT116 cells (a human colon cancer cell line) and the MC38 cells (a mouse colon cancer cell line) (Figure S1B, Supporting Information). In contrast, the numbers of goblet cells (AB/PAS^+^) in the villi of small intestines and the stem cells (SOX9^+^) in the crypts of small intestines and colons were comparable between *Il1f9* ^−/−^ and *Il1f9* ^+/+^ mice (Figure S1A, Supporting Information). These data indicate a proliferative role of IL‐36*γ* in vivo and in vitro.

**Figure 1:**
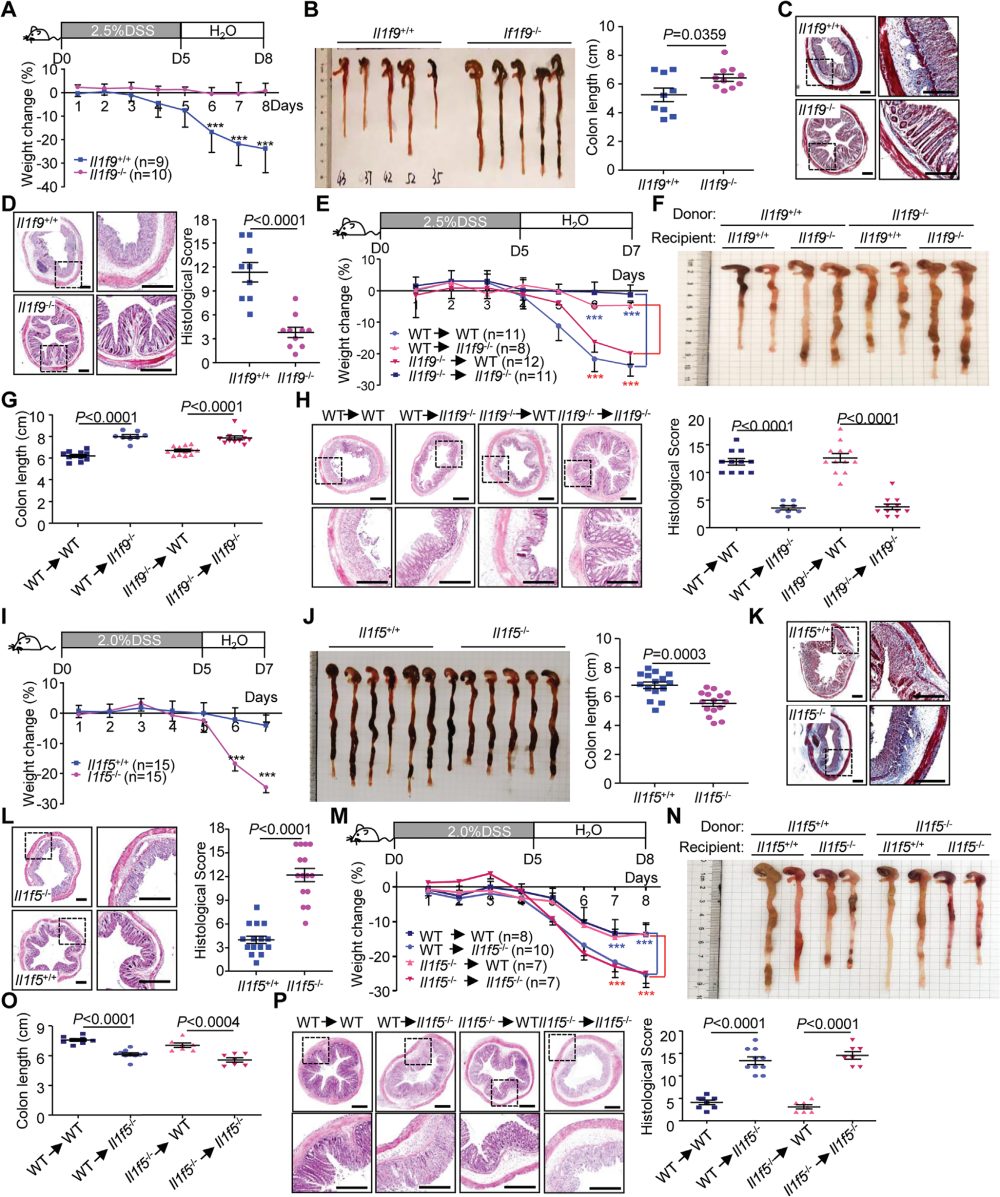
*Il1f9* ^−/−^ and *Il1f5* ^−/−^ mice exhibit hypo‐ and hypersensitivity to DSS‐induced colitis, respectively. A) Body weight change and B) gross morphological change and lengths of colons of *Il1f9* ^+/+^ (*n* = 9) and *Il1f9* ^−/−^ (*n* = 10) mice that were given 2.5% DSS in drinking water for 5 d, followed by normal drinking water for another 3 d. C) Images of Masson's trichrome stained colon sections from *Il1f9* ^+/+^ and *Il1f9* ^−/−^ mice that were treated as in (A). D) Images (left) and pathological scores (right) of HE stained colon sections from *Il1f9* ^+/+^ that were treated as in (A). E) Body weight change of the indicated chimeric mice that were given 2.5% DSS in drinking water for 5 d, followed by normal drinking water for another 2 d. F) Morphological change and G) lengths of colons of the indicated chimeric mice treated as in (E). H) Images (left) and pathological scores (right) of HE stained colon sections of the indicated chimeric mice treated as in (E). I) Body weight change and J) gross morphological change and lengths of colons of *Il1f5* ^+/+^ (*n* = 15) and *Il1f5* ^−/−^ (*n* = 15) mice that were given 2% DSS in drinking water for 5 d, followed by normal drinking water for another 2 d. K) Images of Masson's trichrome stained colon sections from *Il1f5* ^+/+^ and *Il1f5* ^−/−^ mice that were treated as in (I). L) Images (left) and pathological scores (right) of HE stained colon sections of *Il1f5* ^+/+^ and *Il1f5* ^−/−^ mice that were treated as in (I). M) Body weight change of the indicated chimeric mice that were given 2% DSS in drinking water for 5 d, followed by normal drinking water for another 3 d. N) Gross morphological change and O) lengths of colons (O) of the indicated chimeric mice treated as tin (I). P) Images (left) and pathological scores (right) of HE stained colon sections of the indicated chimeric mice treated as in (I). ***P < 0.001 (two‐tailed student's *t*‐test). Graphs show mean ± SEM. Scale bars represent 0.4 mm (C, D, H, K, L, P). Data are combined two (A–H, M–P) or four (I–L) independent experiments.

In DSS‐induced colitis model, the *Il1f9* ^−/−^ mice exhibited increased resistance to weight loss, diarrhea, and colon shortening compared to the controls (**Figure** **1**A,B). Hematoxylin and eosin (HE) and Masson's trichrome staining of the colons showed less leukocyte infiltration, muscle fibers and collagen, and more intact epithelium in the colon from *Il1f9* ^−/−^ mice than *Il1f9* ^+/+^ mice (Figure 1C,D), suggesting a proinflammatory role of IL‐36*γ* in DSS‐induced colitis. Results from quantitative real‐time PCR (qRT‐PCR) and immunohistochemistry (IHC) suggested that IL‐36*γ* was expressed at a low level in colon epithelial cells (ECs) and lamina propria mononuclear cells (LPMC) and upregulated in ECs after DSS treatment (Figure S1C, Supporting Information). Interestingly, irradiated *Il1f9* ^−/−^ mice receiving either *Il1f9* ^+/+^ or *Il1f9* ^−/−^ bone marrow cells were more resistant to DSS‐induced colitis than irradiated *Il1f9* ^+/+^ mice receiving either wild‐type or *Il1f9* ^−/−^ bone marrow cells as monitored by weight loss, colon length, and HE staining of colons (Figure 1E–H), indicating that IL‐36*γ* in nonhematopoietic cells plays a dominant role in promoting DSS‐induced colitis. In this context, our immunohistochemistry staining results suggested predominant expression of IL‐36*γ* in the epithelium (Figure S1D, Supporting Information), which is consistent with recent single‐cell mRNA‐seq data that the levels of *IL1F9* in epithelial cells are higher than those in other types of cells in the tumors from CRC patients and that *IL1F9* is undetectable in CD45^+^ immune cells from the inflamed mucosa of patients with ulcerative colitis.^[^ ^32^, ^33^ ^]^

### 2.2 The *Il1f5* ^−/−^ Mice Exhibit Hypersensitivity to DSS‐Induced Colitis

In contrast to IL‐36*γ* as an agonist for IL‐36R signaling, IL‐36Ra antagonizes IL‐36R signaling by competitively binding to IL‐36R.^[^ ^24^ ^]^ We observed that the number of goblet cells (AB/PAS^+^) in the villi of small intestines and the stem cells (SOX9^+^) in the crypts of small intestines and colons were comparable between *Il1f5* ^−/−^ and *Il1f5* ^+/+^ mice (Figure S1A, Supporting Information). In contrast, the numbers of Paneth cells (lysozyme^+^) were decreased in the small intestines of *Il1f5* ^−/−^ mice compared to *Il1f5* ^+/+^ mice, whereas the proliferative cells (Ki67^+^) in the crypts were slightly increased in the small intestines and colons of *Il1f5* ^−/−^ mice compared to *Il1f5* ^+/+^ mice respectively (Figure S1A, Supporting Information). Consistently, IL‐36Ra antagonized IL‐36*γ*‐induced hyperproliferation of HCT116 cells and MC38 cells (Figure S1B, Supporting Information), indicating that IL‐36*γ* and IL‐36Ra reciprocally regulates intestinal epithelial homeostasis.

We further observed that mice deficient in IL‐36Ra were more sensitive to DSS‐induced colitis than wild‐type controls as monitored by weight loss, diarrhea, colon shortening, and HE and Masson's trichrome staining (Figure 1I–L). Analysis with bone marrow chimeric mice showed that loss of IL‐36Ra in nonhematopoietic cells resulted in hypersensitivity to DSS‐induced colitis (Figure 1M–P). Consistently, IL‐36Ra was predominantly expressed in the epithelium of colon after DSS treatment and immunohistochemistry staining results suggested predominant expression of IL‐36Ra in the epithelium (Figure S1C,D, Supporting Information).^[^ ^33^ ^]^ In contrast, the levels of *Il1rl2* mRNA (encoding IL‐36R) were widely expressed in various types of cells including neutrophils, epithelial cells, T cells and fibroblasts in tumors from CRC patients and in the mucosa of inflamed colon tissues from UC patients.^[^ ^32^, ^33^ ^]^ Collectively, these data suggest that non‐hematopoietic IL‐36*γ* promotes and IL‐36Ra reciprocally inhibits experimental colitis.

### 2.3 IL‐36*γ* and IL‐36Ra Reciprocally Regulate Colon Cancer Progression

**Figure 2:**
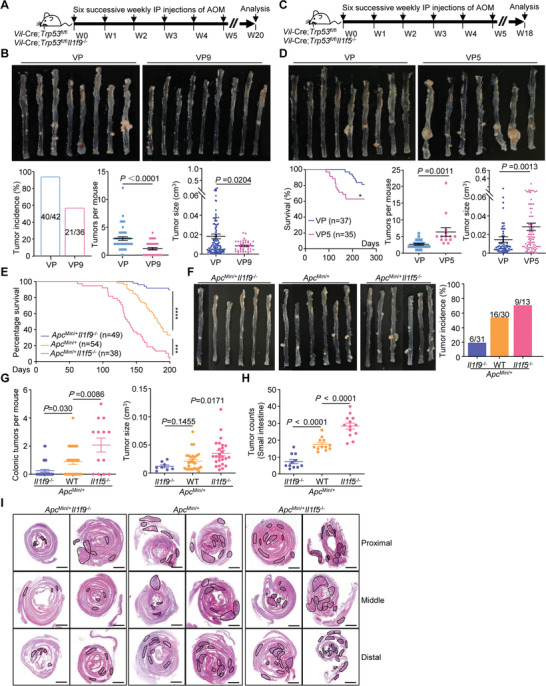
IL‐36*γ* and IL‐36Ra reciprocally regulate tumorigenesis in the colon. A) A scheme of induction of colon cancer with AOM/*Vil*‐Cre;*Trp53* ^fl/fl^ (VP) and AOM/*Vil*‐Cre;*Trp53* ^fl/fl^ *Il1f9* ^−/−^ (VP9) mice. B) Representative images (upper), tumor incidence (lower left), tumor counts (lower middle) and tumor sizes (lower right) in the colons of VP (*n* = 42) and VP9 (*n* = 36) mice that were weekly injected with AOM (10 mg kg^‐1^, intraperitoneally) for six successive weeks and euthanized at the 20th week after initial AOM injection. C) A scheme of induction of colon cancer with AOM/*Vil*‐Cre;*Trp53* ^fl/fl^ (VP) and AOM/*Vil*‐Cre;*Trp53* ^fl/fl^ *Il1f5* ^−/−^ (VP5) mice. D) Images (upper), survival (lower left, VP, *n* = 37 and VP5, *n* = 35), tumor counts (lower middle, VP, *n* = 23 and VP5, *n* = 14) and tumor sizes (lower right) in colons of mice that were weekly injected with AOM (10 mg kg^‐1^, intraperitoneally) for six successive weeks and euthanized at the 20th week after initial AOM injection. E) Survival of *Apc* ^Min/+^ *Il1f9* ^−/−^ (*n* = 49), *Apc* ^Min/+^ (*n* = 54) and *Apc* ^Min/+^ *Il1f5* ^−/−^ (*n* = 38) mice. F) Representative images (left) and tumor incidences (right) in the colons of 5 month old *Apc* ^Min/+^ *Il1f9* ^−/−^ (*n* = 31), *Apc* ^Min/+^ (*n* = 30) and *Apc* ^Min/+^ *Il1f5* ^−/−^ (*n* = 13) mice. G) Tumor counts (left) and tumor volumes (right) in the colons of 5 month old *Apc* ^Min/+^ *Il1f9* ^−/−^ (*n* = 31), *Apc* ^Min/+^ (*n* = 30) and *Apc* ^Min/+^ *Il1f5* ^−/−^ (*n* = 13) mice. H–I) Images of HE stained small intestines (I) and tumor counts (H) in small intestines of 5 month old *Apc* ^Min/+^ *Il1f9* ^−/−^ (*n* = 11), *Apc* ^Min/+^ (*n* = 12) and *Apc* ^Min/+^ *Il1f5* ^−/−^ (*n* = 13) mice. *P < 0.05; ***P < 0.001; ****P < 0.0001 (two‐tailed student's *t*‐test in B, D, G and log‐rank analysis in D,E). Graphs show mean ± SEM. B,D,G,H). Scale bars represent 1 mm (I). Data are combined A–G) four or I,H) two independent experiments.

We next investigated the role of IL‐36*γ* and IL‐36Ra in colon cancer development. In the azoxymethane (AOM)/DSS model of colon cancer, the *Il1f9* ^−/−^ mice exhibited more resistance to weight loss, more rapid recovery, less tumors and smaller tumor sizes in colons than the *Il1f9* ^+/+^ mice (Figure S2A,B and Table S1, Supporting Information). Conversely, the *Il1f5* ^−/−^ mice were more sensitive to the weight loss and developed more tumors with larger sizes in the colon than the *Il1f5* ^+/+^ mice (Figure S2C,D and Table S1, Supporting Information). In the AOM/Vil‐Cre;*Trp53* ^fl/fl^ (VP) model, knockout of IL‐36*γ* significantly reduced tumor incidences and tumor numbers and sizes in colon, whereas knockout of IL‐36Ra led to increased lethality, tumor numbers, and sizes (**Figure** **2**A–D and Table S1, Supporting Information). In addition, the percentages of invasive adenocarcinoma in the colons were decreased or increased by knockout of IL‐36*γ* or IL‐36Ra compared to the controls, respectively (Figure S2E,F, Supporting Information). In a third model of intestinal cancer, ablation of IL‐36*γ* or IL‐36Ra in *Apc* ^Min/+^ mice significantly promoted survival or death of mice, respectively (Figure 2E). Consistently, the tumor incidences, numbers, and sizes were significantly reduced or increased in the colons of *Apc* ^Min/+^ *Il1f9* ^−/−^ mice or *Apc* ^Min/+^ *Il1f5* ^−/−^ mice compared to *Apc* ^Min/+^ mice, respectively (Figure 2F,G and Table S1, Supporting Information). Moreover, the tumor numbers in the small intestine of *Apc* ^Min/+^ mice were also significantly reduced or increased by knockout of IL‐36*γ* or IL‐36Ra, respectively (Figure 2H,I), indicating a protumor role of IL‐36*γ* and an antitumor role of IL‐36Ra in colon cancer progression. We further observed that the levels of *Il1f9* were higher in tumor tissues than in the normal tissues from the same mice or from control mice of the same age (Figure S2G, Supporting Information). Consistently, the levels of IL‐36*γ* were higher in tumor tissues than in the adjacent normal tissues in human CRC biopsies (Figure S2H and Table S2, Supporting Information),^[^ ^34^ ^]^ indicating an upregulation of IL‐36*γ* is accompanied with colon cancer progression. Collectively, these results suggest that IL‐36*γ* promotes and IL‐36Ra reciprocally inhibits gastrointestinal tumorigenesis in multiple mouse models.

### 2.4 IL‐36*γ* and IL‐36Ra Reciprocally Regulate the Expression of Cell–Matrix Molecules during Colonic Inflammation and Tumorigenesis

**Figure 3:**
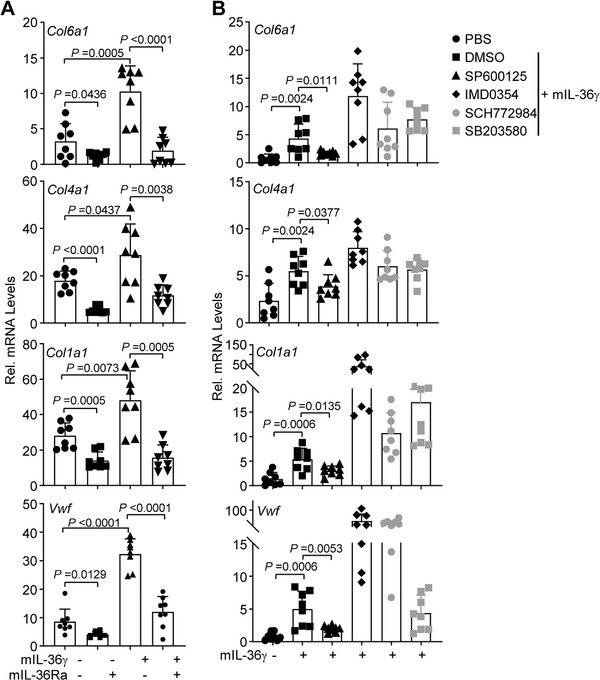
IL‐36*γ* induces upregulation of extracellular matrix genes through the JNK pathway. A) qRT‐PCR analysis of the indicated genes of colon organoids from wild‐type C57BL/6 mice that were left unstimulated or stimulated with IL‐36*γ* (20 ng mL^‐1^) or IL‐36*γ* plus IL‐36Ra (20 ng mL^‐1^) for 6 h. B) qRT‐PCR analysis of the indicated genes of colon organoids from wild‐type C57BL/6 mice that were left unstimulated or stimulated with IL‐36*γ* (20 ng mL^‐1^) plus DMSO, SP600125 (a JNK inhibitor), IMD0354 (an IKK inhibitor), SCH772984 (an ERK inhibitor) or SB203580 (a p38 MAPK inhibitor) for 6 h. A,B) Two‐tailed student's t‐test. A,B) Graphs show mean SEM. A,B) Data are representative of two independent experiments.

RNA sequencing and KEGG pathway analyses suggested that cytokine‐chemokine signaling pathways and cell–matrix adhesion molecules were found differentially expressed with statistical significance in the inflamed colon tissues of DSS‐treated *Il1f9* ^+/+^ and *Il1f9* ^−/−^ mice as well as in the inflamed colon tissues of DSS‐treated *Il1f5* ^+/+^ and *Il1f5* ^−/−^ mice (Figure S3A and Table S3, Supporting Information). Interestingly, however, gene set enrichment analysis (GSEA) and qRT‐PCR analysis suggested that cytokine‐chemokine signaling pathways were similarly downregulated in *Il1f9* ^−/−^ mice and *Il1f5* ^−/−^ mice compared their respective controls (Figure S3B,C and Tables S3 and S4, Supporting Information). In contrast, molecules associated with cell–matrix adhesion and extracellular matrix‐receptor interaction were downregulated in the inflamed colon tissues of *Il1f9* ^−/−^ mice (vs *Il1f9* ^+/+^ mice) and upregulated in the inflamed colon tissues of *Il1f5* ^−/−^ mice (vs *Il1f5* ^+/+^ mice), respectively (Figure S3B,D, Supporting Information). Interestingly, IL‐36*γ* upregulated and IL‐36Ra inhibited the expression of cell‐matrix adhesion genes such as *Col6a1*, *Col1a1*, *Col4a1*, and *Vwf* in mouse colon organoids (**Figure** **3**A). In addition, IL‐36*γ*‐induced upregulation of *Col6a1*, *Col1a1*, *Col4a1*, and *Vwf* in mouse colon organoids was significantly inhibited by SP600125 (a JNK inhibitor) but not by the inhibitors for other kinases including IMD0354 (an IKK inhibitor), SCH772984 (an ERK inhibitor) and SB203580 (a p38 MAPK inhibitor), indicating that JNKs are involved in IL‐36*γ*‐triggered expression of cell‐matrix adhesion genes (Figure 3B). Together with the observations that IL‐36R was expressed in colon epithelium,^[^ ^32^, ^33^, ^35^ ^]^ we conclude that IL‐36*γ* promotes and IL‐36Ra reciprocally inhibits the expression of cell‐matrix adhesion network genes through the JNK pathway in the colon epithelium during colitis.

**Figure 4:**
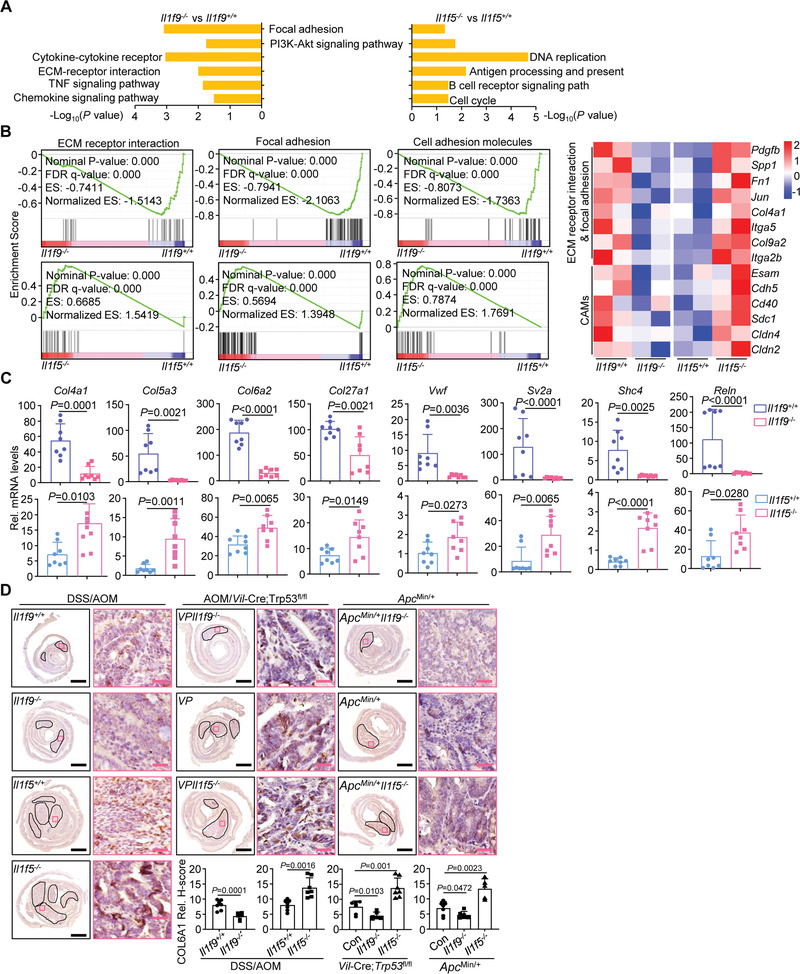
IL‐36*γ* and IL‐36Ra reciprocally regulate the expression of cell‐adhesion matrix molecules during colon tumorigenesis. A) KEGG pathway enrichment analysis of the transcriptome data of colon tumors from *Il1f9* ^+/+^ (*n* = 2) and *Il1f9* ^−/−^ (*n* = 2) mice or *Il1f5* ^+/+^ (*n* = 2) and *Il1f5* ^−/−^ (*n* = 2) mice that were induced colon cancer with the AOM/DSS protocol. B) GSEA analysis (left) and heatmap of selected genes (right) related to extracellular matrix (ECM) receptor interaction, focal adhesion, and cell‐adhesion molecules (CAMs) from the transcriptome data obtained in (A). C) qRT‐PCR analysis of the indicated genes in colon tumors from *Il1f9* ^+/+^ (*n* = 8) and *Il1f9* ^−/−^ (*n* = 8) or *Il1f5* ^+/+^ (*n* = 8) and *Il1f5* ^−/−^ (*n* = 8) mice that were induced colon cancer with the AOM/DSS protocol. D) Immunohistochemistry images (upper) and the intensity quantification (lower) of COL6A1 in the tumors from wild‐type *Il1f9* ^+/+^ (*n* = 7) and *Il1f9* ^−/−^ (*n* = 6) mice or *Il1f5* ^+/+^ (*n* = 7) and *Il1f5* ^−/−^ (*n* = 7) mice that were induced colon cancer with the AOM/DSS protocol (left), VP (*n* = 7), VP9 (*n* = 6), and VP5 (*n* = 7) mice that were induced colon cancer with the AOM protocol (middle), or 5 month old *Apc* ^Min/+^ (*n* = 7), *Apc* ^Min/+^ *Il1f9* ^−/−^ (*n* = 6) and *Apc* ^Min/+^ *Il1f5* ^−/−^ (*n* = 5) mice (right). ES, enrichment score; NES, nonenrichment score; FDR, false discovery rate; FWER, family‐wise error rate. Hypergeometric test in A and B. Two‐tailed student's *t*‐test in C, D. Graph show mean ± SEM. C,D) Scale bars represent 1 mm (black) or 50 µm (red), respectively. B–D) Data are representative of two independent experiments.

We noted that the cytokine and chemokine signaling pathways were similarly downregulated in *Il1f9* ^−/−^ tumors (vs *Il1f9* ^+/+^ tumors) and *Il1f5* ^−/−^ tumors (vs *Il1f5* ^+/+^ tumors) obtained from the AOM/DSS colon cancer model (Figure S4A,B and Tables S4 and S5, Supporting Information). In contrast, cell‐matrix adhesion molecules were differentially regulated with statistical significance in *Il1f9* ^+/+^ versus *Il1f9* ^−/−^ tumors and *Il1f5* ^+/+^ versus *Il1f5* ^−/−^ tumors from mice treated with AOM/DSS (**Figure** **4**A). GSEA and qRT‐PCR analysis confirmed that cell‐matrix network molecules were downregulated and upregulated in *Il1f9* ^−/−^ tumors and *Il1f5* ^−/−^ tumors compared to the controls from multiple mouse models, respectively (Figure 4B,C, Figure S4C and Tables S4 and S5, Supporting Information). In addition, the intensities of COL6A1 staining were lower and higher in *Il1f9* ^−/−^ tumors and *Il1f5* ^−/−^ tumors compared to the controls, respectively (Figure 4D). Consistently with these observations, IL‐36*γ* levels were positively correlated with COL6A1 levels in the human CRC biopsies (Figure S4D and Table S2, Supporting Information). These data together suggest that IL‐36*γ* promotes and IL‐36Ra reciprocally inhibits colon tumorigenesis by remodeling the cell–matrix network. In this context, it has been reported that dysregulated extracellular matrix network creates a tumor‐favoring microenvironment for IBD and CRC progression.^[^ ^19^, ^20^, ^36^ ^]^

### 2.5 IL‐36*γ* Promotes and IL‐36Ra Inhibits Wnt Signaling, Respectively

**Figure 5:**
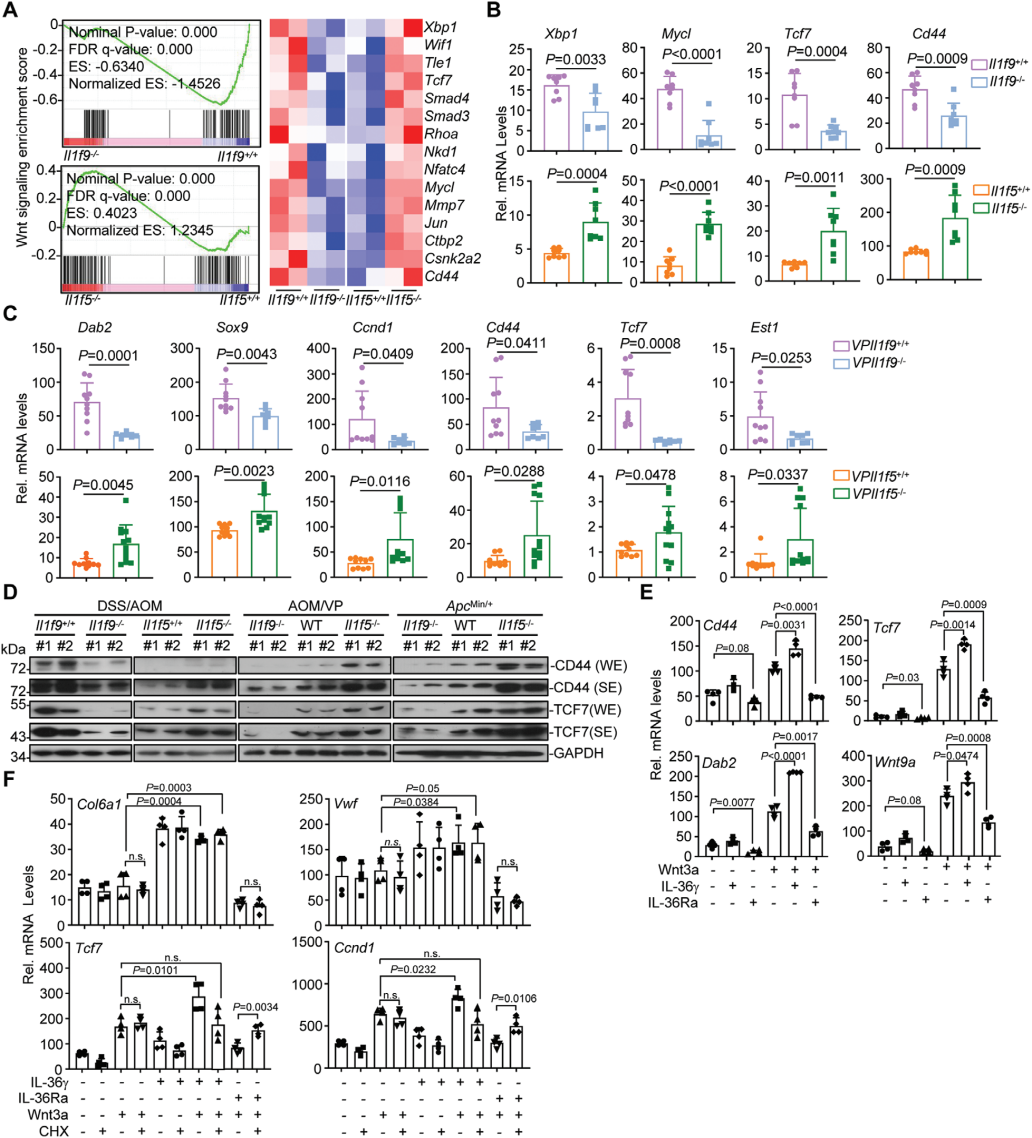
IL‐36*γ* synergizes and IL‐36Ra inhibits Wnt signaling during colitis and colon tumorigenesis. A) GSEA (left) and heatmap (right) of Wnt signaling pathway genes in the transcriptome profiles of colon tumors from *Il1f9* ^+/+^ (*n* = 2) and *Il1f9* ^−/−^ (*n* = 2) mice or from *Il1f5* ^+/+^ (*n* = 2) and *Il1f5* ^−/−^ (*n* = 2) mice that were treated with AOM/DSS protocol. B) qRT‐PCR analysis of the indicated genes in colon tissues from *Il1f9* ^+/+^ (*n* = 8) and *Il1f9* ^−/−^ (*n* = 8) mice or *Il1f5* ^+/+^ (*n* = 8) and *Il1f5* ^−/−^ (*n* = 8) that were treated as in (A). C) qRT‐PCR analysis of the indicated genes in colon tumors of VP (*n* = 10) and VP9 (*n* = 8) or VP (*n* = 10) and VP5 (*n* = 12) mice that were intraperitoneally injected with AOM (10 mg kg^‐1^) every week for 6 successive weeks and rested for 12–14 weeks. D) Immunoblot analysis of CD44, TCF7 or GAPDH in colon tumors from *Il1f9* ^+/+^ and *Il1f9* ^−/−^ (*n* = 2) mice or from *Il1f5* ^+/+^ and *Il1f5* ^−/−^ (*n* = 2) mice treated as in (A) (left), VP, VP9 or VP5 (*n* = 2) mice treated as in (C) (middle), or 5 month old *Apc* ^Min/+^, *Apc* ^Min/+^ *Il1f5* ^−/−^ or *Apc* ^Min/+^ *Il1f9* ^−/−^ (*n* = 2) mice (right). WE, weak exposure; SE, strong exposure. E) qRT‐PCR analysis of the indicated genes in colon organoids (*n* = 4) that were left untreated or stimulated with Wnt3a (100 ng mL^‐1^), IL‐36*γ* (20 ng mL^‐1^), IL‐36Ra (20 ng mL^‐1^), Wnt3a plus IL‐36*γ*, or Wnt3a plus IL‐36Ra for 4 h. F) qRT‐PCR analysis of the indicated genes in colon organoids (*n* = 4) that were left untreated or stimulated with Wnt3a (100 ng mL^‐1^), IL‐36*γ* (20 ng mL^‐1^), IL‐36Ra (20 ng mL^‐1^), Wnt3a plus IL‐36*γ*, or Wnt3a plus IL‐36Ra in the presence or absence of CHX (50 µg mL^‐1^) for 6 h. ES, enrichment score; NES, nonenrichment score; FDR, false discovery rate; FWER, family‐wise error rate. WE, weak exposure; SE, strong exposure. n.s., not significant. Graphs show mean ± SEM. B,C,E,F) Two‐tailed student's *t*‐test in B, C, E, F. B–F) Data are representative of two independent experiments.

The cell–matrix adhesion network and Wnt signaling pathways crosstalk heavily during development.^[^ ^37^, ^38^ ^]^ Interestingly, the Wnt signaling pathway was downregulated in *Il1f9* ^−/−^ colon tissues and tumors and upregulated in *Il1f5* ^−/−^ colon tissues and tumors from DSS‐ or AOM/DSS‐treated mice compared to the controls (**Figure** **5**A,B and Figure S5A,B, Supporting Information). Results from qRT‐PCR and immunoblot analysis confirmed that deficiency in IL‐36*γ* and IL‐36Ra impaired and promoted the expression of downstream genes involved in Wnt signaling in the tumors from AOM/VP and *Apc* ^Min/+^ colon cancer models, respectively (Figure 5C,D and Figure S5C, Supporting Information). Consistently, the levels of IL‐36*γ* were positively correlated with *β*‐Catenin in human CRC biopsies (Figure S5D and Table S2, Supporting Information). Interestingly, IL‐36*γ* alone did not activate the expression of Wnt downstream genes but facilitated Wnt3a‐induced expression of *Dab2*, *Cd44*, *Tcf7*, or *Wnt9a* in colon organoids (Figure 5E), whereas IL‐36Ra impaired basal and Wnt3a‐induced expression of *Dab2*, *Cd44*, *Tcf7*, or *Wnt9a* (Figure 5E). In addition, treatment of cycloheximide (CHX) impaired IL‐36*γ*‐mediated potentiation of and IL‐36Ra‐mediated suppression of Wnt3a‐induced expression of *Tcf7* or *Ccnd1* (Figure 5F). These data together suggest essential roles of IL‐36*γ* and IL‐36Ra in facilitating and antagonizing Wnt signaling in a manner dependently on protein synthesis, respectively.

### 2.6 Inhibition of IL‐36*γ* Maturation Alleviates Colitis and Colon Tumorigenesis

**Figure 6:**
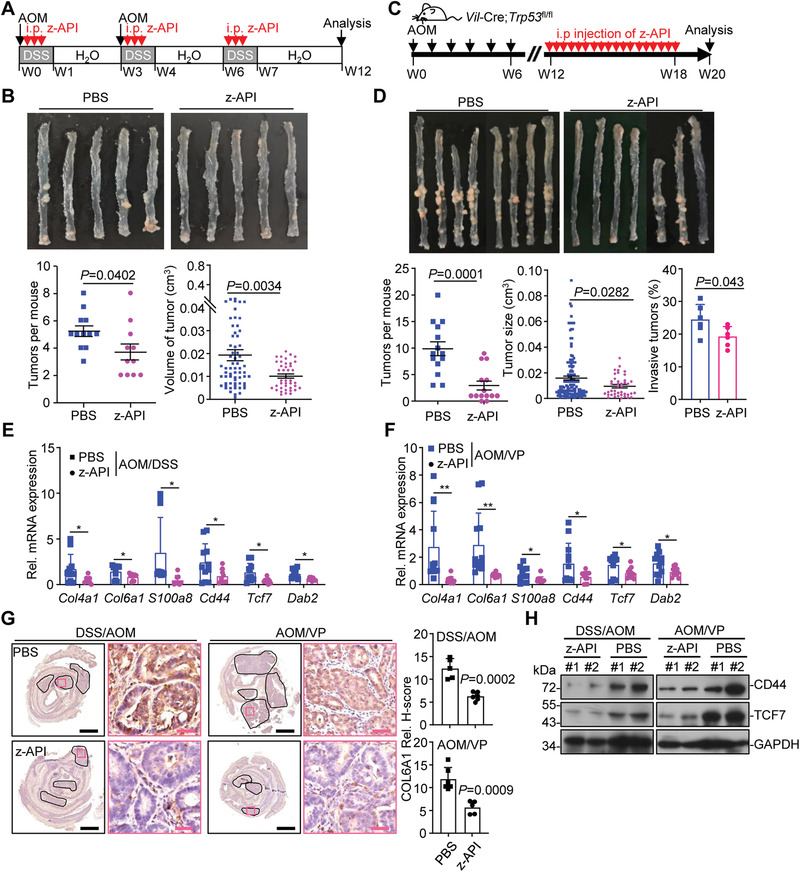
Inhibition of IL‐36*γ* maturation impairs colon tumorigenesis. A) A scheme of z‐API treatment during the induction of AOM/DSS colon cancer. The mice were induced colon cancer with the AOM/DSS (2.5% DSS) protocol and intraperitoneally injected daily with z‐API (100 µg) or PBS during the three rounds of DSS treatment. B) Images (upper), tumor counts (lower left), and tumor volumes (lower right) of colons from wild‐type C57BL/6 mice treated as in (A) (*n* = 12 or 11 for PBS or z‐API, respectively). C) A scheme of z‐API treatment during the induction of AOM/*Vil*‐Cre;*Trp53* ^fl/fl^ model. *Vil*‐Cre;*Trp53* ^fl/fl^ mice were weekly injected with AOM (10 mg kg^‐1^) for six successive weeks, followed by intraperitoneal injection of z‐API (100 µg) or PBS every other day from the 12th to the 18th week after the initial AOM injection. The mice were euthanized at the 20th week after the initial AOM injection. D) Representative images (upper), tumor counts (lower left) (n = 14 for PBS or z‐API), tumor sizes (lower middle) (n = 14 for PBS or z‐API), and the invasive tumor percentages (lower right) (n = 6 for PBS or z‐API) of colons from *Vil*‐Cre;*Trp53* ^fl/fl^ mice treated as in (D). E) qRT‐PCR analysis of the indicated genes in the colon tumors from mice treated as in (A) (*n* = 9 or 11 for PBS or z‐API, respectively). F) qRT‐PCR analysis of the indicated genes in the colon tumors from mice treated as in (D) (*n* = 12 for PBS or z‐API). G) IHC images (left) and quantification analysis (right) of colon sections from mice treated as in (A) (*n* = 6 for PBS and *n* = 6 for z‐API) or in (D) (*n* = 6 for PBS and *n* = 6 for z‐API). (H) Immunoblot analysis of the indicated proteins in colon tumors of mice treated as in (A, left, *n* = 2 for PBS or z‐API) or in (D, right, *n* = 2 for PBS or z‐API). *P < 0.05; **P < 0.01 (two‐tailed student's *t*‐test in B, C, E–G). Graphs show mean ± SEM in (B,D,E–G). Scale bars represent 1 mm (black) or 50 µm (red), respectively. Data are combined two (B,D,E,F) independent experiments or representative of two (G,H) independent experiments.

Elastase‐mediated cleavage at the N‐termini of IL‐36 cytokines is critical for the maturation and activation, which can be blocked by the z‐Ala‐Pro‐Ile (z‐API) peptide.^[^ ^39^, ^40^ ^]^ Intraperitoneal injection of z‐API alleviated the symptoms of colitis in wild‐type mice but not in *Il1f9* ^−/−^ mice (Figure S6, Supporting Information), indicating that IL‐36*γ* is the major target of z‐API in the DSS colitis model. We further found that the AOM/DSS‐treated mice or the AOM‐treated VP mice exhibited reduced numbers and sizes of colon tumors after intraperitoneal injection of z‐API compared to PBS (**Figure** **6**A–D and Table S1, Supporting Information). In addition, the percentages of invasive adenocarcinoma in the z‐API‐treated VP mice were significantly decreased compared to the PBS‐treated VP mice (Figure 6D). The expression of cell–matrix adhesion molecules and Wnt downstream genes was also inhibited in mice receiving z‐API compared to the controls (Figure 6E–H), indicating that intervention of IL‐36*γ* maturation effectively alleviates colitis, colon tumorigenesis, and tumor invasiveness.

### 2.7 Neutralizing IL‐36*γ* Inhibits Colitis and Colon Tumorigenesis

To more specifically block IL‐36*γ* in vivo, we generated a polyclonal antibody against IL‐36*γ* by immunizing rabbits followed by affinity purification^[^ ^41^ ^]^ (Figure S7A, Supporting Information). The antibody inhibited IL‐36*γ*‐ but not IL‐36*α*‐, IL‐36*β*‐ or TNF‐induced activation of NF‐*κ*B reporter in HEK293‐mIL‐36R cells or expression of downstream genes in colon organoids (Figure S7B–D, Supporting Information). In addition, intraperitoneal injection of anti‐IL‐36*γ* significantly alleviated the inflammation and downregulated the expression of cell‐matrix adhesion molecules and Wnt downstream genes in colons of wild‐type mice but not in those of *Il1f9* ^−/−^ mice after DSS treatment (Figure S7E–K, Supporting Information), indicating high selectivity and efficacy of the obtained IL‐36*γ* antibody in vitro and in vivo.

**Figure 7:**
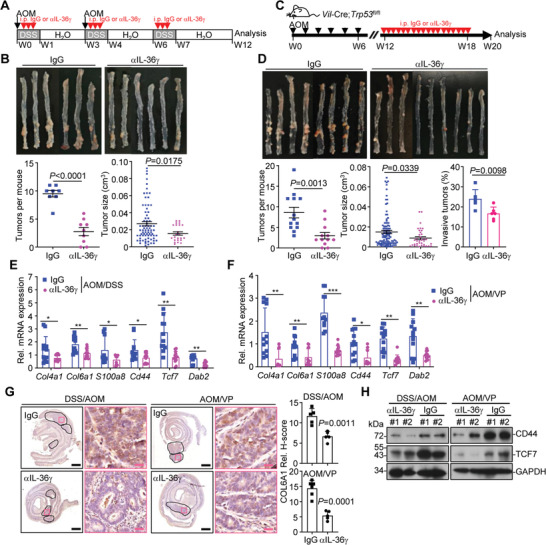
Neutralization of IL‐36*γ* alleviates colitis and colon cancer. A) A scheme of treatment with *α*IL‐36*γ* (100 µg, intraperitoneally injected daily during 2.5% DSS treatment) or control IgG (upper) of wild‐type C57BL/6 mice that were treated with AOM/DSS protocol. B) Images (upper), tumor counts (lower left), tumor volumes (lower right) of colons from wild‐type C57BL/6 mice treated as in (A) (*n* = 9 or 8 for *α*IL‐36*γ* or IgG, respectively). C) A scheme of treatment with *α*IL‐36*γ* (100 µg, intraperitoneally injected every other day from the 12th to the 18th week after initial AOM injection) or control IgG of *Vil*‐Cre;*Trp53* ^fl/fl^ mice that were intraperitoneally injected with AOM (10 mg kg^‐1^) weekly for 6 successive weeks. D) Representative images (upper), tumor counts (lower left)(n = 12 or 13 for αIL‐36γ or IgG, respectively), tumor volumes (lower middle)(n = 12 or 13 for αIL‐36γ or IgG, respectively), and the invasive tumor percentages (lower right) (n = 6 for αIL‐36γ or IgG) of colons from *Vil*‐Cre;*Trp53* ^fl/fl^ mice treated as in (C). E) qRT‐PCR of the indicated genes in colon tumors from mice treated in (A) (*n* = 12 for *α*IL‐36*γ* or IgG, respectively). F) qRT‐PCR of the indicated genes in colon tumors from mice treated in (C) (*n* = 12 for *α*IL‐36*γ* or IgG, respectively). G) Images of immunohistochemistry (left images) and quantification analysis (right graphs) of the indicated protein in colon tumors from mice treated as in (A, *n* = 5 for IgG and *α*IL‐36*γ* groups) or (C, *n* = 6 or 5 for IgG or *α*IL‐36*γ*, respectively). H) Immunoblot analysis of related proteins in colon tumors of mice treated as in (A or C). *P < 0.05; **P < 0.01; ***P < 0.001 (two‐tailed student's *t*‐test in B, D, E, F). Graphs show mean ± SEM. B,D,E,F) Scale bars represent 1 mm (black) or 50 µm (red), respectively. Data are combined two (A, B) or three (D, E) independent experiments or representative of two (F, G) independent experiments.

We next examined the effects of anti‐IL‐36*γ* in colon tumorigenesis in multiple colon cancer models. In the *Apc* ^Min/+^ intestinal cancer model, injection of anti‐IL‐36*γ* significantly improved the survival of *Apc* ^Min/+^ mice and inhibited the tumorigenesis and tumor development in the colon and the small intestine compared to injection of the control IgG (Figure S8A–C, Supporting Information). In addition, the tumor incidences, numbers, and sizes in the colons from AOM/DSS‐treated mice and AOM‐treated VP mice were significantly reduced by anti‐IL‐36*γ* compared with control IgG (**Figure** **7**A–D and Table S1, Supporting Information). Moreover, the percentages of invasive adenocarcinoma in the colons of AOM‐treated VP mice were significantly decreased by injection of anti‐IL‐36*γ* (Figure 7D). Consistently, the expression of cell‐matrix adhesion molecules and Wnt downstream genes in the colon tumors was impaired by anti‐IL‐36*γ* (Figure 7E–G and Figure S8D, Supporting Information). These data together suggest that blocking IL‐36*γ* effectively inhibits experimental colitis and colon cancer development.

## 3 Discussion

The incidences of IBD and CRC have been increasing in developing countries, which cause a major public health problem.^[^ ^1^, ^20^ ^]^ Mechanistic understanding of IBD and CRC would contribute to the development of therapeutic intervention strategies and improve the prognosis and life quality of patients. In this study, we have demonstrated that IL‐36*γ* promoted and IL‐36Ra reciprocally inhibited experimental colitis and colon cancer development in multiple murine models. In addition, targeting IL‐36*γ* by maturation blockade or neutralization significantly inhibited colitis and colon cancer development, indicating IL‐36*γ* as a potential intervention target for the treatment of IBD and CRC (Figure S8E, Supporting Information). IL‐36*γ* and IL‐36Ra were upregulated in the epithelium but not in the lamina propria lymphocytes of inflamed colons from mice treated with DSS. Consistently, analysis with bone marrow chimeric mice suggested that IL‐36*γ* and IL‐36Ra from nonhematopoietic cells played dominant roles during DSS‐induced colitis. Recent scRNA sequencing studies have suggested that IL‐36*γ* and IL‐36Ra are expressed at higher levels in epithelial cells than in other types of cells from tumors of CRC patients,^[^ ^33^ ^]^ indicating that immune cells are not a primary source of IL‐36*γ* and IL‐36Ra in colon tumors. It is thus likely that nonhematopoietic IL‐36*γ* and IL‐36Ra crosstalk with IL‐36R‐expressing cells to regulate the progression of inflammation and tumorigenesis in the colon. Further investigations with IL‐36*γ* and IL‐36Ra conditional knockout mice are required to fully characterize the contribution of IL‐36*γ* and IL‐36Ra of different cellular sources to the progression of colitis and colon cancer.

It has been well recognized that IL‐36*γ* agonizes and IL‐36Ra antagonizes IL‐36R‐mediated activation of NF‐*κ*B and induction of proinflammatory cytokines and chemokines in cells in vitro.^[^ ^23^, ^42^ ^]^ However, the levels of total and phosphorylated (p) p65 were comparable between the control and the IL‐36*γ* KO or the IL‐36Ra KO tumors (data not shown), indicating that knockout of IL‐36*γ* or IL‐36Ra had minimal effect on the activation or expression of NF‐*κ*B in the tumor tissues. It is not surprising, as the in vivo environment for tumorigenesis (chronic, mixed milieu, weeks to months) is much more complicated than the in vitro environment for cell cultures (acute, pure cytokine stimuli, minutes to hours).

Although the activation status of NF‐*κ*B is comparable between the IL‐36*γ* KO or the IL‐36Ra KO tumors and the controls, the expression levels of cytokine and chemokines were similarly downregulated in *Il1f9* ^−/−^ and *Il1f5* ^−/−^ inflammatory colon tissues or colon tumors compared to the controls. The exact mechanisms behind the phenomenon are unclear and the results in current study indicate more sophisticated regulation of cytokine networks in vivo than in vitro. Nonetheless, these findings have several implications. First, NF‐*κ*B is not the only determinant for proinflammatory cytokine expression in vivo, at least in the colitis and colon cancer models downstream of IL‐36*γ* and IL‐36Ra. In this context, IL‐36*γ* also activates and IL‐36Ra antagonizes the MAPK signaling pathways (such as Jun) that synergize with NF‐*κ*B for the induction of proinflammatory cytokines.^[^ ^42^, ^43^ ^]^ Second, IL‐36*γ* and IL‐36Ra do not reciprocally regulate the expression of proinflammatory cytokines in the in vivo tumor models as they do in the in vitro cultures. It is thus unlikely that IL‐36*γ* and IL‐36Ra regulate colitis and colon cancer progression through modulating the expression of proinflammatory cytokines. Third, downregulation of cytokine and chemokine signaling pathways might not be a prerequisite for inhibition of IBD or CRC progression. Previously studies have shown that higher levels of proinflammatory cytokines in the inflamed colon tissues have been observed with resistance or sensitivity to DSS‐induced colitis in mice.^[^ ^31^, ^44^ ^]^ In addition, mice deficient in IL‐1*β* or TNF are hyper‐sensitive to DSS‐induced colitis and exhibit tissue repair defect after DSS treatment,^[^ ^45^, ^46^ ^]^ and *IL18* genetic polymorphisms known to reduce *IL18* mRNA and protein levels are positively associated with the susceptibility to Crohn's disease,^[^ ^47^ ^]^ suggesting that dysregulation of cytokine networks in colon inflammation and cancer is likely a consequence rather than a cause for disease progression. In this context, clinical trials with blockades targeting proinflammatory cytokines for IBD or CRC have shown limited appreciable efficacy,^[^ ^48^, ^49^ ^]^ although elevated cytokine and chemokine signaling networks have been noted in the inflamed colon tissues of IBD patients or in the tumor tissues of CRC patients.^[^ ^50^, ^51^ ^]^

It has been reported that *Il1rl2* ^−/−^ mice exhibit reduced disease severity in an acute DSS‐induced model of colitis,^[^ ^52^ ^]^ whereas the same mice are sensitive to DSS‐induced colitis and exhibit impaired IL‐22 production and mucosal healing in the colon.^[^ ^29^, ^30^ ^]^ Considering that IL‐36R is widely expressed in various types of cells including epithelial cells, fibroblasts, T cells, and neutrophils,^[^ ^33^ ^]^ the discrepancies among these studies might be due to different functions of IL‐36R in distinct types of cells. We found that IL‐36*γ* promoted expression of an array of genes involved in cell–matrix interaction in colon organoids, indicating the involvement of IL‐36R signaling in remodeling of ECM and cell‐matrix interaction in nonimmune cells. In this context, a recent report has shown that mice deficient in IL‐36R develop less severe colitis and fibrosis than the wild‐type mice after chronic DSS treatment.^[^ ^35^ ^]^ In contrast, IL‐36R signaling in T cells promotes IL‐22 production and clearance of enteropathogenic bacteria in the gut, whereas IL‐36R signaling in macrophages promotes M1 polarization and clearance of *Legionella pneumophila* in the lung.^[^ ^53^, ^54^ ^]^ Therefore, IL‐36R plays proinflammatory roles in non‐immune cells by remodeling ECM and fibrosis and anti‐inflammatory roles in immune cells by promoting bacterial clearance and tissue repair, respectively. On the other hand, IL‐36R mediates both active and suppressive signaling upon binding to different IL‐36 cytokines. IL‐36Ra treatment actively inhibited the basal expression of an array of genes involved in cell‐matrix interaction and such an inhibition did not require de novo protein synthesis, indicating that IL‐36Ra triggers an active suppressive signal in addition to out‐compete the binding of IL‐36*α*/*β*/*γ* to IL‐36R. Future studies are required to elucidate the mechanisms of such an active suppression signaling downstream of IL‐36Ra. Nonetheless, current data support the notion that the balance between agonist and antagonist stimulation of IL‐36R in different types of cells determines the net outcome of the phenotypes of IL‐36R deficiency in DSS‐induced colitis.

It has been previously shown that ectopic expression of IL‐36*γ* in B16F10 melanoma cells and 4T1 breast tumor cells results in activation of type 1 immune responses and tumor regression.^[^ ^55^ ^]^ Interestingly, ectopic expression of IL‐36*α* in basal keratinocytes leads to cutaneous inflammation which is exacerbated by knockout of IL‐36Ra in mice,^[^ ^25^ ^]^ indicating a proimmune role of IL‐36 cytokines in the skin.^[^ ^56^ ^]^ In our study, we observed that the cytokine and chemokine expression and signaling pathways were similarly downregulated in the IL‐36*γ* KO and the IL‐36Ra KO colitis tissues and colon tumors compared to their respective controls. In contrast, the cell–matrix adhesion and Wnt signaling pathways were inhibited in the IL‐36*γ* KO colon tumors and potentiated in the IL‐36Ra KO colon tumors, respectively, indicating that IL‐36*γ* and IL‐36Ra reciprocally regulate cell‐matrix and Wnt signaling but not cytokine‐ and chemokine‐related immune signaling in the colon. In addition, we have recently shown that IL‐36*γ* and IL‐36Ra reciprocally regulate non‐small cell lung cancer progression by modulating GSH homeostasis and oxidative stress‐induced cell death.^[^ ^41^ ^]^ Therefore, it is possible that IL‐36 cytokines exert pro‐ or anti‐tumor activities in different types of cancers by modulating distinct signaling pathways. In this context, the expression levels of IL‐36*γ* are dramatically decreased in skin cutaneous melanoma compared to the adjacent normal skin tissues and are higher in colon and rectum adenocarcinoma tissues than in the adjacent normal tissues.

In contrast to similar downregulation of cytokine and chemokine signaling in *Il1f9* ^−/−^ and *Il1f5* ^−/−^ inflamed colon tissues or colon tumors, the ECM, cell‐matrix interaction and cell adhesion pathways exhibited downregulation and upregulation in *Il1f9* ^−/−^ and *Il1f5* ^−/−^ inflamed colon tissues or colon tumors compared to the respective controls. Specifically, knockout or neutralization of IL‐36*γ* or administration of IL‐36Ra significantly inhibited the expression of cell‐matrix adhesion molecules such as *Vwf*, *Col4a1*, and *Col6a1*, whereas knockout of IL‐36Ra had opposite effects in DSS colitis and colon cancer models, indicating that the agonistic and antagonistic IL‐36R signaling reciprocally controls extracellular matrix network for pro‐ and anti‐inflammation and tumorigenesis in the gastrointestinal system, respectively. In this context, it has been demonstrated that aberrant collagen network promotes CRC development by modulating the behaviors of tumor cells, tumor‐associated fibroblasts, and endothelial cells.^[^ ^19^, ^20^ ^]^ Collectively, these data support the notion that normalizing the cell–matrix microenvironment would be more effective than normalizing cytokine and chemokine signaling as therapeutic strategy for IBD and colon cancer.^[^ ^12^ ^]^

The ECM network not only provides physical scaffolds for tissue organization but also controls cellular behaviors by directly triggering intracellular signaling or by indirectly modulating the microenvironment.^[^ ^57^, ^58^ ^]^ It has been demonstrated that the crosstalk between the cell–matrix network and Wnt signaling is critical for development and genes involved in Wnt signaling pathways exhibit hypermutation or hyperactivation in CRC.^[^ ^37^, ^59^ ^]^ Our data suggested that IL‐36*γ* did not upregulate the expression of Wnt downstream genes but synergized with Wnt3a to induce Wnt target genes in a manner dependently on protein synthesis. In addition, IL‐36Ra alone suppressed basal and Wnt3a‐induced expression of Wnt target genes and CHX treatment impaired IL‐36Ra‐mediated suppression of Wnt target genes after Wnt3a stimulation. Considering that IL‐36*γ* induced and IL‐36Ra inhibited the expression of cell–matrix adhesion molecules, it is likely that IL‐36*γ* and IL‐36Ra reciprocally regulate colon tumorigenesis by directly modulating the cell–matrix adhesion network to indirectly regulate Wnt signaling. Future studies are required to further characterize the crosstalk between the cell–matrix network and Wnt signaling during disease progression. Taken together, the identification of IL‐36*γ* and IL‐36Ra in regulating colitis and colon cancer development provides potential therapeutic strategies for treatment of IBD and CRC.

## 4 Experimental Section

### Mice

*Il1f9* ^+/−^ (#032395‐UCD) and *Il1f5* ^+/−^ (#032393‐UCD) mice were obtained from the Mutant Mouse Resource & Research Center (MMRRC) and were crossed with C57BL/6 mice (GemPharmatech Co., Ltd, Nanjing, China) for at least 6 generations before subsequent studies. *Trp53* ^fl/fl^ (#008462) mice were purchased from Jackson Lab. Wildtype C57BL/6 mice, *Villin*‐Cre (#T000142) and *Apc* ^Min/+^ (#T001457) mice were from GemPharmatech Co., Ltd (Nanjing, China). *Il1f9* ^+/+^ and *Il1f9* ^−/−^ (from *Il1f9* ^+/−^ breeders) or *Il1f5* ^+/+^and *Il1f5* ^−/−^ (from *Il1f5* ^+/−^ breeders) littermates were used throughout the study. *Villin*‐Cre;*Trp53* ^fl/fl^, *Villin*‐Cre;*Trp53* ^fl/fl^ *Il1f9* ^−/−^, *Villin*‐Cre;*Trp53* ^fl/fl^ *Il1f5* ^−/−^mice were bred independently. Male *Apc* ^Min/+^, *Apc* ^Min/+^ *Il1f9* ^−/−^, and *Apc* ^Min/+^ *Il1f5* ^‐/‐^ mice were bred with wild‐type female C57BL/6 mice, *Il1f9* ^−/−^, or *Il1f5* ^‐/‐^ mice, respectively. No statistical methods were used to predetermine sample size. For all experiments presented in this study, age‐ and sex‐matched mice were used and the sample sizes were large enough to determine statistically significant effects. The control and experimental groups of mice were cohoused and randomly allocated to different treatment. All mice were housed in the specific pathogen‐free animal facility at Wuhan University with a 12 h dark/12 h light cycle and fed with standard food and water. All animal experiments were in accordance with protocols approved by the Institutional Animal Care and Use Committee of Wuhan University.

### Human Material

The colorectal tumor samples and adjacent normal tissues were collected during November of 2016 to November of 2017 at the Department of Pathology of Zhongnan Hospital of Wuhan University (Table S2, Supporting Information). The tumor tissues were fixed with 4% paraformaldehyde and embedded in paraffin blocks. The tissue array was prepared as previously described and subject to immunohistochemistry.^[^ ^31^, ^60^ ^]^ For human samples, informed consent was obtained from all patients. Protocols in this study were approved by the Institutional Review Committees of Zhongnan Hospital of Wuhan University, and the Medical Ethic Committee of the School of Medicine, Wuhan University.

### DSS‐Induced Colitis and Colon Cancer Models

The experiments were performed as previously described.^[^ ^31^ ^]^ For the DSS‐induced colitis model, mice were administered the indicated dosages of DSS dissolved in sterile water for 5 d, followed by regular water for 1–3 d. The colons were harvested and washed with PBS for various analyses. For the AOM/DSS colorectal tumor model, cohoused experimental mice were intraperitoneally injected with azoxymethane (AOM, 10 mg kg^‐1^) (#A5486, Sigma), followed by treatment of 2.5% DSS drinking water for 7 d and regular water for 14 d. This cycle was repeated three times and mice were sacrificed at the end of the 12th week. For the *Villin*‐Cre;*Trp53* ^fl/fl^ colon cancer model, 8 week old mice were intraperitoneally injected with AOM (10 mg kg_‐1_) once a week for six successive weeks. At the 20th week after the initial AOM injection, the colons of mice were harvested, washed of feces with cold PBS, and slit open longitudinally to count tumors. The mice were monitored for survival or sacrificed at 18–20 weeks after the last injection of AOM for analysis. For the Apc^Min/+^ colon cancer model, mice were fed with normal food and water for 5 months. The colons and intestines were harvested, washed, and slit open longitudinally for various analyses. All animal experiments were in accordance with protocols approved by the Institutional Animal Care and Use Committee of Wuhan University.

### Tumor Count and Measurement

The colons together with a ruler were imaged for subsequent tumor count and measurement. For tumor count, two students independently counted the tumors in each colon for twice and an average of the counted numbers was calculated as #tumors per mouse. For tumor measurement, the diameter of each tumor was scaled with the referenced ruler and the volume of the tumor was calculated with the formula 4*π*R^3^/3. The size and diameter of colon tumors were listed in Table S1 (Supporting Information). The invasive adenocarcinoma and adenoma were characterized as previously described.^[^ ^61^ ^]^

### Measurement of Cell Proliferation

The E‐plates in the xCELLigence real‐time Cell Analyzer (RTCA) system (Agilent) were adopted to continuously monitor the proliferation of cells in cultures. In brief, the HCT116 cells and the MC38 cells (5000 cells per well in 200 µL medium) were seeded on the E‐Plates and cultured in an incubator (37 °C, 5% CO_2_). Twelve hours later, the cells were treated with IL‐36*γ* (20 ng mL^‐1^) or IL‐36*γ* plus IL‐36Ra (20 ng mL^‐1^). The proliferation of the cells was continuously monitored and quantitated by the RTCA system 16 (Agilent).

### mRNA Transcriptome Sequencing

*Il1f9* ^+/+^ and *Il1f9* ^−/−^ mice were administered 2.5% DSS dissolved in sterile water for 5 d, followed by regular water for 2 d. *Il1f5* ^+/+^ and *Il1f5* ^−/−^ mice were administered 2% DSS dissolved in sterile water for 5 d, followed by regular water for 2 d. The colons were flushed with PBS to clear feces and slit open longitudinally. The distal colon tissues (0.5 cm in length, about 0.5 cm away from the anus) were washed in PBS and homogenized in 2 mL TRIzol (Invitrogen). Colon tumors from *Il1f9* ^+/+^ and *Il1f9* ^−/−^ mice or *Il1f5* ^+/+^ and *Il1f5* ^−/−^ mice that were given AOM/DSS treatment were washed in PBS and homogenized in 2 mL TRIzol (Invitrogen). Total RNAs were prepared and the qualities of RNAs were determined by agarose gel electrophoresis and spectrophotometer analysis. Poly(A) mRNA was subsequently purified from 10 µg total RNA using NEBNext Oligo d(T)25 Magnetic Beads Isolation Module. The first‐strand cDNA was synthesized with NEBNext RNA first‐strand synthesis module. NEBNext Ultra II nondirectional RNA second‐strand synthesis module was used for the synthesis of the complementary strand of first‐strand cDNA. The resulted double‐stranded DNA was purified and Vazyme TruePrep DNA Library Prep Kit V2 was used to prepare libraries followed by sequencing on an Illumina Hiseq X Ten platform with 100 bp paired‐end reads strategy (Novogene Co. Ltd, Beijing). Quality control of mRNA‐seq data was performed by using Fatsqc and low‐quality bases were trimmed by Cutadapt. All RNA‐seq data were mapped to the mouse genome (mm9) by TopHat (version 2.1.1) and allowed maximum 2 mismatch per read. Gene expression level was calculated by Cufflinks with default parameters and normalized by FPKM. Gene ontology and KEGG pathway enrichment analysis were performed using DAVID (https://david.ncifcrf.gov). The transcriptome data and the gene sets used for GSEA were included in Tables S3–S5 (Supporting Information).

### Bone Marrow Transfer

Bone marrow cells were isolated from the femur of donor mice, lysed with RBC lysis buffer, and washed with PBS for three times. The recipient mice were irradiated with 8 Gy and immediately injected with the isolated bone marrow cells through tail vein (10^6^ cells per mouse). Induction of colitis was performed at the eighth week after bone marrow transfer.

### Colon Organoid Culture

Mouse primary intestine or colon organoid was generated and cultured as described previously.^[31]^ Briefly, colon tissues were vigorously shaken in 2 × 10^‐3^ m EDTA for 1 h at 4 °C, followed by removing the EDTA solution. Subsequently loosened crypts were prepared by pipetting the solution up and down with 15 mL PBS through a 10 mL pipette for 8–10 times followed by 1 min rest. The supernatants containing the crypt suspension were transferred into a new 50 mL centrifuge tube and the precipitants were resuspended with 15 mL PBS and pipetted up and down for repeated collection of crypts. FBS was added to the crypt suspensions until a final concentration of 10%. The supernatants containing crypts were centrifuged at 300 *g* for 5 min. Pelleted crypts were resuspended in 2 mL conditional medium with growth factors (CMGF advanced DMEM/F12), and collected by centrifugation at 130 *g* for 5 min at 4 °C. Crypts were finally suspended in Matrigel (Corning, Bedford, USA), and placed in the center of a well of a 24‐well plate (40 µL per well). After the Matrigel had solidified (15 min at 37 °C), crypts were cultured in culture medium (#06005, Stem Cell) at 37 °C with 5% CO_2_. The culture medium was refreshed every 2–3 d until experiments.

### Hematoxylin‐Eosin Staining and Masson's Trichrome Staining Analysis

Colon tissues from mice with DSS‐induced colitis or colonic tumors were fixed in 4% paraformaldehyde and embedded in paraffin blocks. The paraffin blocks were sectioned (5 µm) for HE staining (Beyotime Biotech) or Masson's trichrome staining (Leagene) followed by coverslipped. Images were acquired using an Aperio VERSA 8 (Leica) multifunctional scanner. The pathogenic scores of the inflamed colons were evaluated by combining the disease activity index (DAI) score and histopathological score. DAI score was assessed for each animal as a cumulative score for the severity of colitis, according to the stool consistency (score: 0, normal; 1, soft and shaped; 2, loose stools; 3, diarrhea), rectal bleeding (score: 0, normal; 1 and 2, bloody stool; 3, gross bleeding), and body weight loss (score: 0, none; 1, 1%–5%; 2, 5%–10%; 3, 10%–15%; 4, >15%). Histopathological changes were analyzed as a cumulative score, based on epithelial damage (0, normal morphology; 1, loss of goblet cells; 2, loss of goblet cells in large areas; 3, loss of crypts; 4, loss of crypts in large areas), and inflammatory cell infiltration (0, no infiltration; 1, infiltration around crypt bases; 2, infiltration spreading to muscularis mucosa; 3, extensive infiltration in the muscularis mucosa with abundant edema; 4, infiltration spreading to submucosa).

### Immunohistochemistry (IHC) Assays

IHC assays were performed as previously described.^[^ ^31^, ^60^ ^]^ In brief, the paraffin sections were deparaffinized with xylene and immersed in 100%, 85%, and 70% ethanol. The antigen retrieval was performed with 0.5 × 10^‐3^ m EDTA (pH8.0) by heating in a microwave oven for 30 min. The sections were incubated with antibodies, followed by counterstaining with hematoxylin. Sections were incubated with a biotinylated secondary antibody followed by ABC reagent (UltraSensitiveTM SP IHC Kit, MXB). Histological analysis for inflammation, epithelial hyperplasia, and tumorigenesis was performed by a board‐certified pathologist (Dr. Zhi‐Gao Xu). Signals were imaged with an Aperio VERSA 8 (Leica) multifunctional scanner and quantified with the software Image‐Pro Plus 6.0.

### Isolation of Colon Epithelial Cells and Lamina Propria Mononuclear Cells

Colons were flushed with PBS to clear feces and slit open longitudinally, cut into 0.5 cm pieces, incubated in epithelial cell isolation buffer (5 mL RPMI 1640, 500 µL FBS, 200 µL 0.5 × 10^‐3^ m EDTA, 20 µL 1 × 10^‐3^ m DTT, 20 µL 50 µg mL gentamicin) and shaken at 180 rpm at 37 °C for 30 min. The epithelial cell isolation buffer was filtered through a sterile nylon membrane and the flow‐through was centrifuged at 1000 rpm at 4 °C for 5 min and saved as epithelial cells. The colon tissues on the sterile nylon membrane were washed with PBS (10 mL) two times, incubated with lamina propria mononuclear cell buffer (5 mL RPMI 1640, 500 µL FBS, 50 µL 1.5 mg mL^‐1^ type IV collagenase, 20 µL 10 µg mL^‐1^ DNase I) and shaken at 180 rpm at 37 °C for 30 min. The lamina propria mononuclear cell buffer was filtered through a sterile nylon membrane and the flow‐through was centrifuged at 1000 rpm at 4 °C for 5 min. The precipitants were resuspended in 40% percoll–PBS buffer (3 mL, GE healthcare, 17‐0891‐09) and 80% percoll–PBS buffer (3 mL) was added at the bottom of the 40% percoll–PBS buffer tube. The tube was centrifuged at 2000 rpm at room temperature for 20 min. The intermediate phase containing LPMCs was collected and washed with PBS followed by various analyses.

### Immunoblot and qRT‐PCR Assays

Immunoblot and qRT‐PCR assays were performed as previously described.^[^ ^31^, ^62^, ^63^ ^]^ The colon tissues or tumors were homogenized with NP‐40 lysis buffer (150 × 10^‐3^ m NaCl, 1 × 10^‐3^ m EDTA, 1% nonidet P‐40) supplemented with proteinase and phosphatase inhibitors (Biotool). The lysates were cleared by centrifuge for 10 min at 4 °C. The supernatants were quantified and loaded to 10%–12% sodium dodecyl sulfate–polyacrylamide gel (SDS‐PAGE) for electrophoresis and transferred onto nitrocellulose membranes. Blocking was performed in 5% BSA in TBS for 1 h, and membranes were incubated with primary antibodies overnight at 4 °C. Membranes were incubated with horseradish peroxidase‐conjugated secondary antibody for 1 h, and proteins were visualized by using ECL substrate. Primary antibodies used in this study were listed in Table S6 (Supporting Information).

Total RNA was extracted from distal colons of mice after colitis induction, colon tumor tissues, or organoids using TRIzol (Invitrogen). The total RNAs (10 µg) were reverse‐transcribed into first‐strand cDNAs with All‐in‐One cDNA Synthesis SuperMix (50 µL) (Biotool) in the presence of spermine (Sigma, 55513, 10 ng µL^‐1^). Gene expression was examined with a Bio‐Rad CFX Connect system by a fast two‐step amplification program with 2× SYBR Green Fast qPCR Master Mix (Biotool). The *c*_t_ value obtained for each gene was normalized to that of the gene encoding *β*‐actin. Gene‐specific primers were listed in Table S7 (Supporting Information).

### Purification of IL‐36*γ*

The complementary DNA encoding mouse IL‐36*γ* was obtained by reverse‐transcription PCR with mRNA from DSS‐treated colon tissues. The cDNAs were amplified and cloned into the pET30c vector and confirmed by sequencing. The generated plasmids were transformed into *E. coli* (Rosseta2) competent cells that were cultured in 10 mL LB medium at 37 °C for overnight. These cells were further cultured in 1 L LB medium at 37 °C for 3–4 h until an OD600 value of 0.6–0.8 followed by cooling on ice. IPTG (0.3 × 10^‐3^ m) were added into the medium and cultured at 18 °C for 24 h. The cells were harvested and lysed with Tris‐NaCl buffer (25 × 10^‐3^ m Tris, 150 × 10^‐3^ m NaCl, pH = 8.0) by high pressure crushing. The cell lysates were centrifuged at 12 000 *g* for 10 min at 4 °C and the His‐TEV‐tagged IL‐36*γ* proteins in the supernatants were purified with Ni^2+^ affinity chromatography and eluted with iminazole. The untagged IL‐36*γ* proteins were obtained by mixing His‐TEV‐tagged IL‐36*γ* proteins with His‐TEV protease (kindly provided by Dr. Lei Yin, Wuhan University) at 4 °C for overnight followed by Ni^2+^ affinity chromatography and size exclusion chromatography. The activity of the obtained IL‐36*γ* proteins was determined by luciferase reporter assay.

### Immunization of IL‐36*γ* and Antibody Purification

The New Zealand rabbits were subcutaneously immunized with IL‐36*γ* (400 µg) emulsified in competent Freud's adjuvant at week 0. At weeks 1, 3, and 6, the rabbits were subcutaneously immunized with IL‐36*γ* (200 µg) emulsified in incomplete Freud's adjuvant. At weeks 0, 4, and 7, ≈2 mL blood was collected for ELISA analysis. At week 9, the rabbits were intravenously injected with IL‐36*γ* (400 µg). At week 10, the rabbits were euthanized and the blood was obtained to isolate the antisera by centrifuge of 1000 g for 5 min at 4 °C. The antisera were cleared by further centrifuge at 20 000 *g* for 20 min at 4 °C and diluted by 10 times volume of the binding buffer (0.1 m phosphate buffer, 0.15 m NaCl, pH 8.0). The diluted antisera were loaded to the pre‐equilibrated Protein A column followed by washing with the binding buffer for three times. The anti‐IL‐36*γ* IgG was eluted with the elution buffer (0.2 m sodium citrate, pH 3.0) and dialyzed with PBS. The neutralization activity of *α*IL‐36*γ* was determined with NF‐*κ*B luciferase reporter assay. The control rabbit IgG was purified from unimmunized rabbits.

### IL‐36*α*, IL‐36*β*, IL‐36*γ*, IL‐36Ra, and TNF Stimulation

These cytokines were dissolved in PBS (5 mg mL^‐1^) and aliquoted for stock at ‐80 °C until use. The cytokines were diluted with PBS and added into the culture medium of cells or colon organoids (50 µL for each well) with a final concentration of 20 ng mL^‐1^. The cells or colon organoids were harvested for qRT‐PCR or luciferase reporter assays after 3–8 h stimulation, respectively. These cytokines used in this study were listed in Table S6 (Supporting Information).

### Treatment of Mice with z‐API or Anti‐IL‐36*γ*

z‐API was dissolved in PBS (10 mg mL^‐1^ and 10 mg mL^‐1^, respectively) and aliquoted for stock at ‐80 °C until use. z‐API (100 µg, 20 µL) or anti‐IL‐36*γ* (100 µg) was diluted in 100–200 mL PBS and intraperitoneally injected into mice once a day during the induction of DSS‐induced colitis. For mice treated with AOM/DSS cycles, z‐API (100 µg, 20 µL) or anti‐IL‐36*γ* (100 µg) diluted in 100–200 mL PBS was intraperitoneally injected into mice once a day for 7 successive days during the DSS treatment. For VP mice treated with AOM, z‐API (100 µg, 20 µL) or anti‐IL‐36*γ* (100 µg) diluted in 100–200 mL PBS was intraperitoneally injected into mice every other day for 7 successive weeks from the 12th week through the 18th week after the initial injection of AOM. For *Apc* ^Min/+^ mice, anti‐IL‐36*γ* (100 µg) diluted in 100–200 mL PBS was intraperitoneally injected into mice (8 week old) every other day for six successive weeks. The mice were left for survival observation or sacrificed at 20 week old.

### Analysis of scRNA Sequencing Data

For analysis of CRC samples, the raw counts matrix of scRNA sequencing data of CRC tissues was kindly provided by Dr. Zemin Zhang (Peking University).^[^ ^33^ ^]^ The downstream analysis was performed with R packages Seurat (version 3.2.3). Highly variable genes were used for principal‐component analysis in Seurat. After principal‐component analysis, significant principal components were identified by using the knee in the scree plot. Only scores from these significant principal components were used as the input to nearest‐neighbor based clustering (“FindClusters,” resolutions = 0.1) and t‐SNE (“RunTSNE,” dims = 1:10). For analysis of ulcerative colitis samples, the CellRanger (v2.0)‐processed count files and metadata were downloaded from single‐cell portal (https://singlecell.broadinstitute.org/single_cell) under the accession number SCP259.^[^ ^32^ ^]^ The clusters were annotated according to the respective marker genes as described.^[^ ^32^, ^33^ ^]^

### Transfection and Reporter Assays

HEK293 cells were transiently transfected with NF‐*κ*B‐driven firefly luciferase reporter (100 ng), TK‐Renilla luciferase reporter (20 ng) and mIL‐36R (100 ng) using standard calcium phosphate precipitation. Twenty hours after transfection, the cells were stimulated with IL‐36*α*, IL‐36*β*, IL‐36*γ* (20 ng mL^‐1^) or TNF (5 ng mL^‐1^) in the presence or absence of anti‐IL‐36*γ* for 8 h followed by luciferase assays with a dual‐specific luciferase reporter kit (Promega). The activity of firefly luciferase was normalized by that of Renilla luciferase to obtain relative luciferase activity

### Statistics Analysis

Differences between experimental and control groups were tested using two‐tailed Student's t‐test. The number of repeats for each experiment was indicated in the respective figure legends. N in the figure legends indicates the number of mice or replicates in the experiments. P values less than 0.05 were considered statistically significant. For animal survival analysis, the Kaplan‐Meier method was adopted to generate graphs, and the survival curves were analyzed with log‐rank analysis. Prism 8.3.0 was used to generate graphs and perform statistical analysis.

## Conflict of Interest

H.W. and Y.L. are founders and share‐holders of Yurogen Biosystems LLC (Wuhan). J.X. and J.C. are employees of Yurogen Biosystems LLC (Wuhan).

## Author Contributions

W.Y., H.‐P.D., and P.W. contributed equally to this work. B.Z. and D.L. designed and supervised the study. W.Y. and H.‐P.D. performed the major experiments. P.W. performed bioinformatics analysis of the sequencing data and the sc‐RNA sequencing data; Z.‐G.X., B.X., and D.L. collected and analyzed the UC and CRC samples. J.X., J.C., H.W., and Y.L. performed immunization and antibody purification assays. B.Z. and D.L. wrote the paper. All the authors analyzed data.

## Supporting information

Supporting Information

Supporting Table 1

Supporting Table 2

Supporting Table 3

Supporting Table 4

Supporting Table 5

Supporting Table 6

Supporting Table 7

Supporting Figure 1

## Data Availability

The data that support the findings of this study are available from the corresponding author upon reasonable request.
