# Supplementary material for: IL‐36γ and IL‐36Ra Reciprocally Regulate Colon Inflammation and Tumorigenesis by Modulating the Cell–Matrix Adhesion Network and Wnt Signaling
Source: Adv Sci (Weinh). 2022 Feb 4;9(10):2103035. doi: 10.1002/advs.202103035 (PMC8981487; doi:10.1002/advs.202103035)
Supplement: Supplementary file 1 — Supporting Information [file ADVS-9-2103035-s009.pdf]

ADVANCED SCIENCE
Open Access

Supporting Information

for *Adv. Sci.*, DOI 10.1002/advs.202103035

IL-36γ and IL-36Ra Reciprocally Regulate Colon Inflammation and Tumorigenesis by Modulating the Cell–Matrix Adhesion Network and Wnt Signaling

Wei Yang, Hong-Peng Dong, Peng Wang, Zhi-Gao Xu, Jiahuan Xian, Jiachen Chen, Hai Wu, Yang Lou, Dandan Lin and Bo Zhong**

ADVANCED SCIENCE
Open Access

Supporting Information

for *Adv. Sci.*, DOI: 10.1002/advs.202103035

IL-36γ and IL-36Ra reciprocally regulate colon inflammation and tumorigenesis by modulating the cell-matrix adhesion network and Wnt signaling

Wei Yang, Hong-Peng Dong, Peng Wang, Zhi-Gao Xu, Jiahuan Xian, Jiachen Chen, Hai Wu, Yang Lou, Dandan Lin,, Bo Zhong**

Supplementary Figure 1

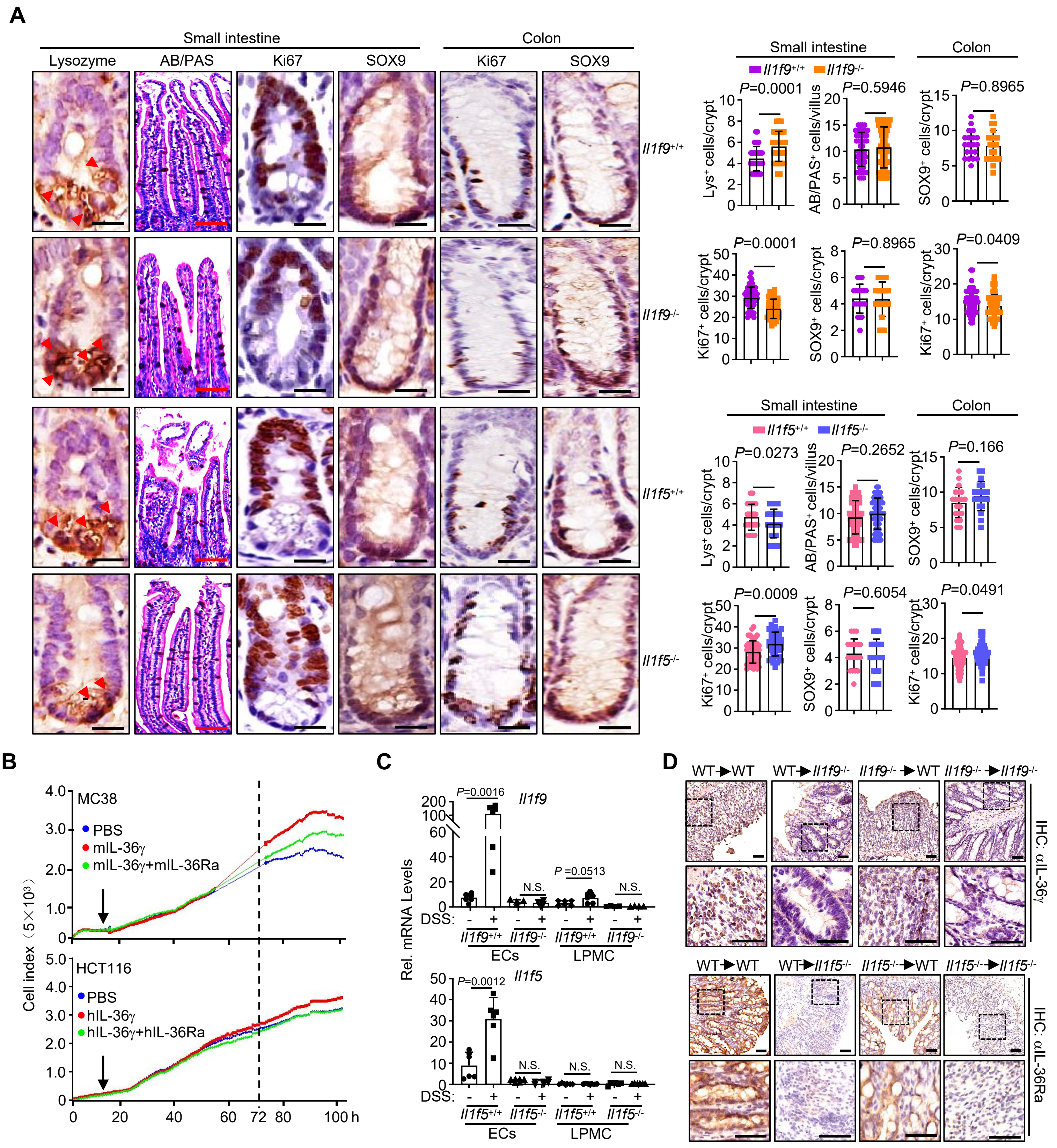

Supplementary Figure 1 IL-36γ is upregulated in inflammatory colon tissues.

(A) IHC (left images) and quantification analysis (right graphs) of Lysozyme (n= 50 crypts from three mice), AB/PAS (n= 50 villi from three mice) staining of crypts or villi of small intestines, Ki67 (n= 50 crypts from three mice) or SOX9 (n= 20 crypts from three mice) staining of crypts of small intestines and colons of *Il1f9*$^{+/+}$ and *Il1f9*$^{-/-}$ mice (n=3) or *Il1f5*$^{+/+}$ and *Il1f5*$^{-/-}$ mice (n=3).

(B) Proliferation analysis of the MC38 (upper graph) cells and the HCT116 (lower graph) cells that were left unstimulated or stimulated with IL-36γ (20 ng/ml) or IL-36γ plus IL-36Ra (20 ng/ml).

(C) qRT-PCR analysis of the *Il1f9* in colon epithelial cells and LPMCs from *Il1f9*$^{+/+}$ (n=6) and *Il1f9*$^{-/-}$ (n=4) mice that were treated with or without 2.5% DSS for 5 days (upper graph), or of *Il1f5* in colon epithelial cells and LPMCs from *Il1f5*$^{+/+}$ (n=6) and *Il1f5*$^{-/-}$ (n=6) mice (n=6) that were treated with or without 2% DSS for 5 days (lower graph).

(D) IHC analysis of IL-36γ (upper) or IL-36Ra (lower) in the colons from the indicated bone marrow chimeric mice after colitis induction.

Graphs show mean ± SEM. (A, C). Two-tailed student's *t*-test. Scale bars represent 100 μm (A, red), 20 μm (A, black) or 50 μm (D), respectively. Data are representative of two independent experiments.

Supplementary Figure 2

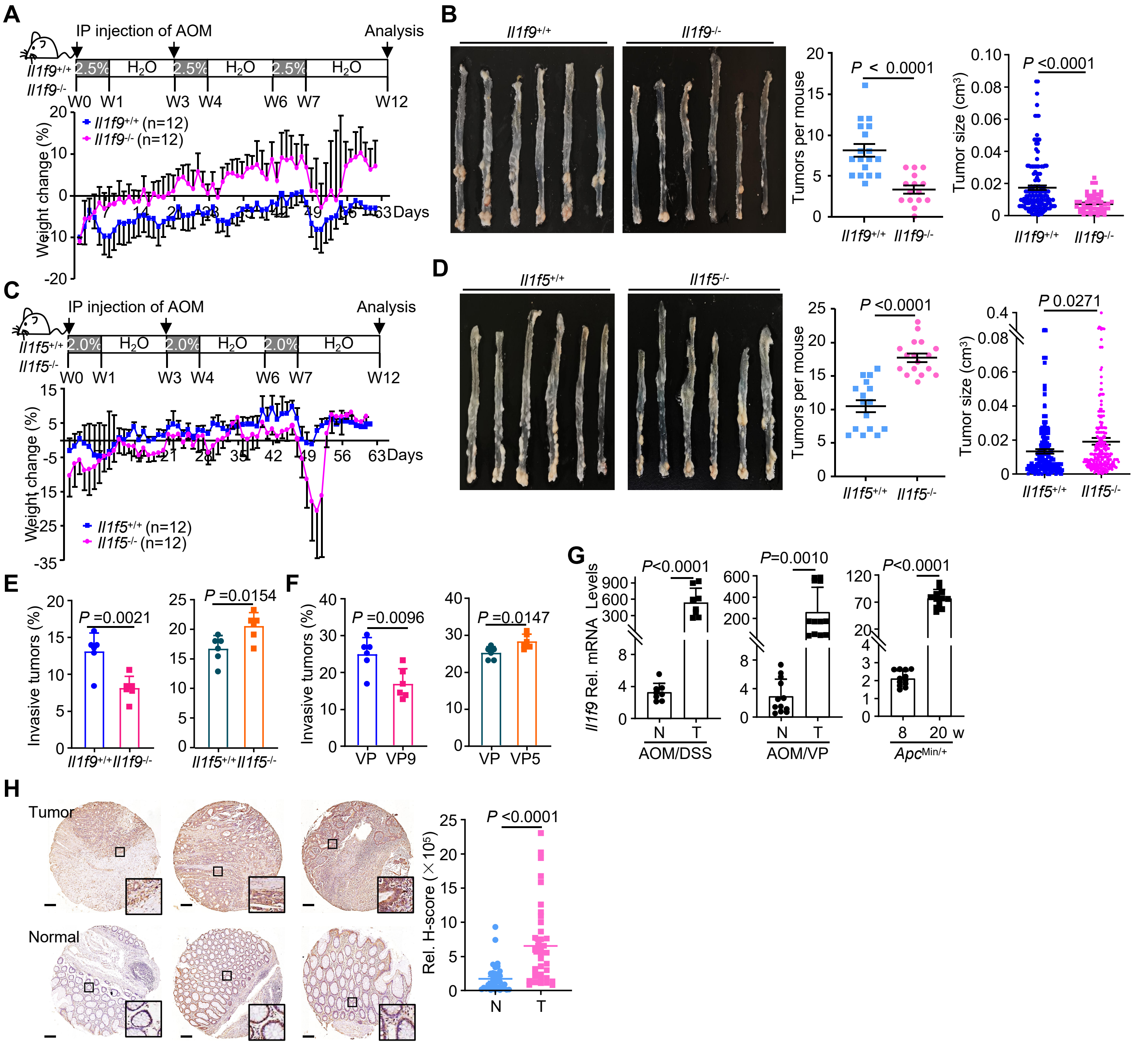

Supplementary Figure 2 IL-36γ promotes and IL-36Ra inhibits colon tumorigenesis.

(A) A scheme of AOM/DSS-induced (2.5% DSS) colon cancer (upper) and weight change (lower) of *Il1f9*$^{+/+}$ (n=12) and *Il1f9*$^{-/-}$ (n=12) mice.

(B) Images of (left), tumor counts (meddle) and tumor volumes (right) in the colons from *Il1f9*$^{+/+}$ (n=18) and *Il1f9*$^{-/-}$ (n=15) mice that were treated as in (A).

(C) A scheme of AOM/DSS-induced (2% DSS) colon cancer (upper) and weight change (lower) of *Il1f5*$^{+/+}$ (n=12) and *Il1f5*$^{-/-}$ (n=12) mice.

(D) Images of (left), tumor counts (middle) and tumor volumes (right) in the colons from *Il1f5*$^{+/+}$ (n=16) and *Il1f5*$^{-/-}$ (n=17) mice that were treated as in (C).

(E) Percentages of invasive adenocarcinoma in the colons of *Il1f9*$^{+/+}$ (n=6) and *Il1f9*$^{-/-}$ (n=6) mice (left), *Il1f5*$^{+/+}$ (n=6) and *Il1f5*$^{-/-}$ (n=6) mice (right) that were treated by the AOM/DSS protocol in (A) and (C), respectively.

(F) Percentages of invasive adenocarcinoma in the colons of VP (n=6) and VP9 (n=6) mice(right), VP (n=6) and VP5 (n=6) mice (left) that were treated with AOM.

(G) qRT-PCR of *Il1f9* in the colon tumors versus the normal colon tissues from the AOM/DSS (n=8 for normal or tumor, respectively), AOM/VP (n=12 for normal or tumor, respectively) or *Apc*$^{\mathrm{Min}/+}$ mice (n=12 for normal or tumor, respectively).

(H) Images (left) and quantification analysis (right) of IHC with anti-IL-36γ in human CRC biopsies and the adjacent normal colon tissues (n=36).

Graphs show mean ± SEM. Two-tailed student's *t*-test. Scale bars represent 0.4 mm (H). Data are combined two (A, C) or three (B, D) independent experiments or representative of two independent experiments (G).

Supplementary Figure 3

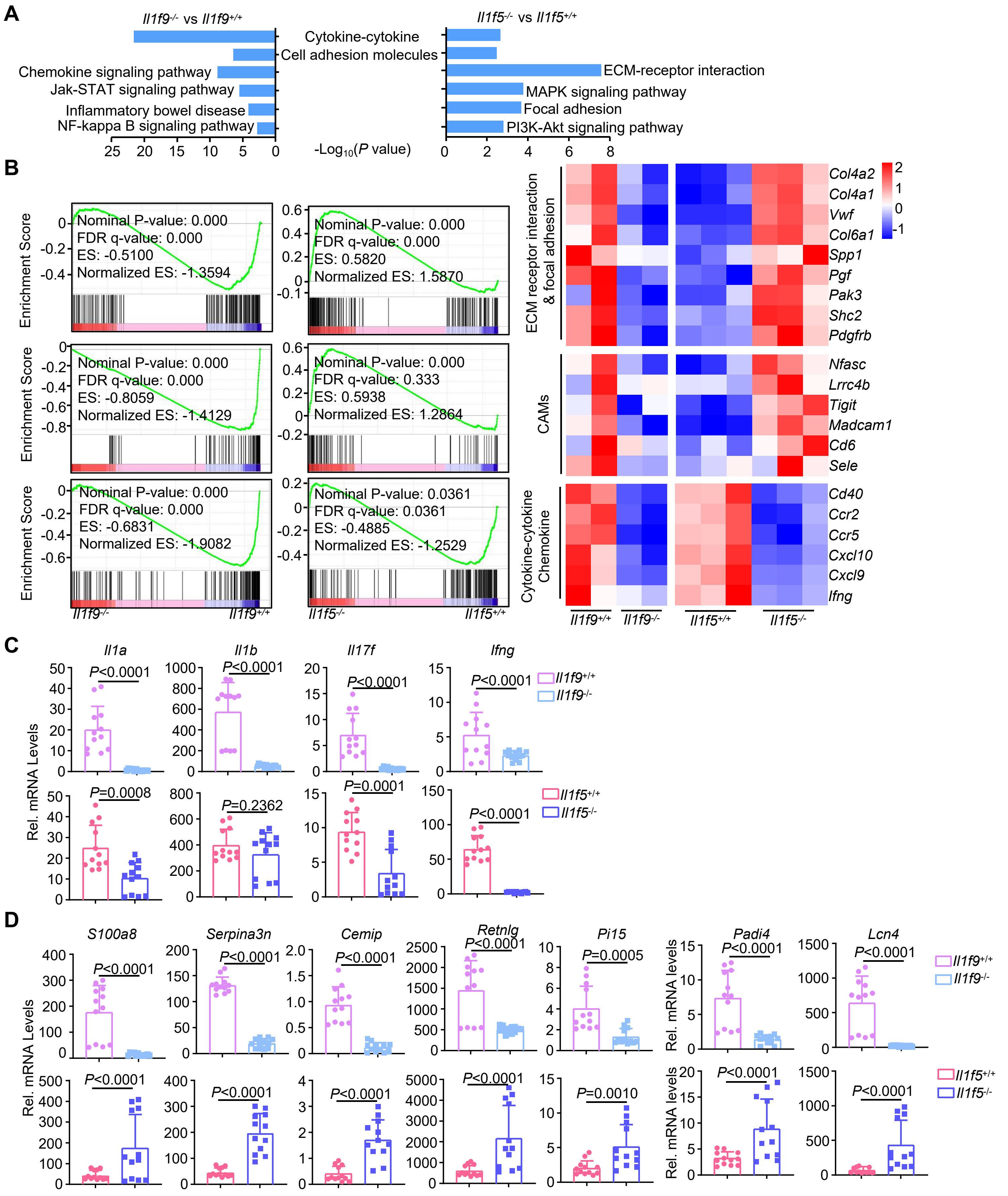

Supplementary Figure 3 IL-36γ and IL-36Ra reciprocally regulate the expression of cell-adhesion matrix molecules during DSS-induced colitis.

(A) KEGG pathway enrichment analysis of the transcriptome of colon tissues from *Il1f9*$^{+/+}$ (n=2) and *Il1f9*$^{-/-}$ (n=2) mice that were given 2.5% DSS in drinking water for 5 d, followed by normal drinking water for another 2 d (left) or from *Il1f5*$^{+/+}$ (n=3) and *Il1f5*$^{-/-}$ (n=3) mice that were given 2% DSS in drinking water for 5 d, followed by normal drinking water for another 2 d (right).

(B) GSEA analysis (left) and heatmap (right) of the selected genes related to extracellular matrix (ECM) receptor interaction, focal adhesion, cell-adhesion molecules, and cytokine-cytokine and chemokine from *Il1f9*$^{+/+}$ (n=2) and *Il1f9*$^{-/-}$ (n=2) mice or *Il1f5*$^{+/+}$ (n=3) and *Il1f5*$^{-/-}$ (n=3) mice that were treated as in (A).

(C-D) qRT-PCR analysis of the indicated genes of colon tissues from *Il1f9*$^{+/+}$ (n=12) and *Il1f9*$^{-/-}$ (n=12) or *Il1f5*$^{+/+}$ (n=12) and *Il1f5*$^{-/-}$ (n=12) mice that were treated as in (A).

ES, enrichment score; NES, non-enrichment score; FDR, false discovery rate; FWER, family-wise error rate. Hypergeometric test (A) or two-tailed student's *t*-test (C, D). Graphs show mean ± SEM. (C, D). Data are representative of two independent experiments (C, D).

Supplementary Figure 4

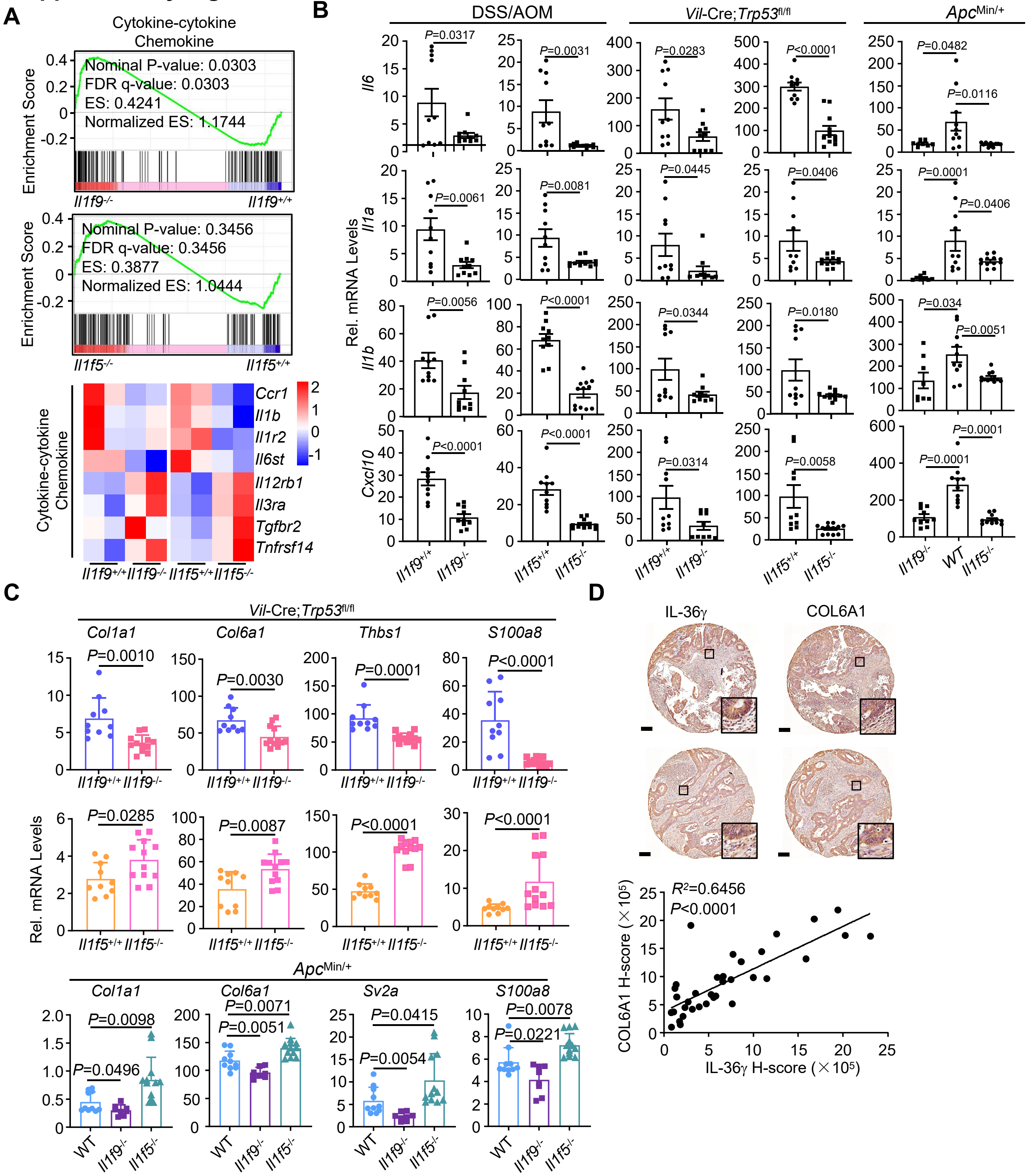

Supplementary Figure 4 IL-36γ and IL-36Ra reciprocally regulate the expression of cell-adhesion matrix molecules during colon tumorigenesis.

(A) GSEA analysis (upper) and heatmap (lower) of the selected genes related to cytokine-cytokine receptor and chemokine in tumors from *Il1f9*$^{+/+}$ (n=2) and *Il1f9*$^{-/-}$ (n=2) mice or *Il1f5*$^{+/+}$ (n=2) and *Il1f5*$^{-/-}$ (n=2) mice that were induced colon cancer with the AOM/DSS (2.5% DSS for *Il1f9*$^{+/+}$ and *Il1f9*$^{-/-}$ mice and 2.5% DSS for *Il1f5*$^{+/+}$ and *Il1f5*$^{-/-}$ mice, respectively) protocol.

(B) qRT-PCR analysis of *Il6*, *Il1a*, *Il1b* or *Cxcl10* of colon tumors from *Il1f9*$^{+/+}$ (n=10) and *Il1f9*$^{-/-}$ (n=10) or *Il1f5*$^{+/+}$ (n=10) and *Il1f5*$^{-/-}$ (n=10) mice that were induced colon cancer with the AOM/DSS protocol (left), *Vil*-Cre;*Trp53*$^{fl/fl}$ (n=10) and *Vil*-Cre;*Trp53*$^{fl/fl}$*Il1f9*$^{-/-}$ (n=10) or *Vil*-Cre;*Trp53*$^{fl/fl}$(n=10) and *Vil*-Cre;*Trp53*$^{fl/fl}$*Il1f5*$^{-/-}$ (n=12) mice that were induced colon cancer with weekly i.p. injection of AOM for 6 successive weeks (middle), or 5-month-old *Apc*$^{Min/+}$ (n=6), *Apc*$^{Min/+}$*Il1f9*$^{-/-}$ (n=6) and *Apc*$^{Min/+}$*Il1f5*$^{-/-}$ (n=6) mice(right).

(C) qRT-PCR analysis of the indicated genes of colon tumors from *Vil*-Cre;*Trp53*$^{fl/fl}$ (n=6) and *Vil*-Cre;*Trp53*$^{fl/fl}$*Il1f9*$^{-/-}$ (n=6) or *Vil*-Cre;*Trp53*$^{fl/fl}$(n=6) and *Vil*-Cre;*Trp53*$^{fl/fl}$*Il1f5*$^{-/-}$ (n=6) mice that were induced colon cancer with weekly i.p. injection of AOM for 6 successive weeks (upper and middle graphs), or 5-month-old *Apc*$^{Min/+}$ (n=10), *Apc*$^{Min/+}$*Il1f9*$^{-/-}$ (n=10) or *Apc*$^{Min/+}$*Il1f5*$^{-/-}$ (n=12) mice (lower graphs).

(D) IHC staining (left) and Person correlation analysis (right) of IL-36γ and COL6A1 in human CRC biopsies (n=36).

ES, enrichment score; NES, non-enrichment score; FDR, false discovery rate; FWER, family-wise error rate. Graphs show mean ± SEM. (B, C). Two-tailed student's *t*-test in B, C. Scale bars represent 0.4 mm (D). Data are representative of two independent experiments (B, C).

Supplementary Figure 5

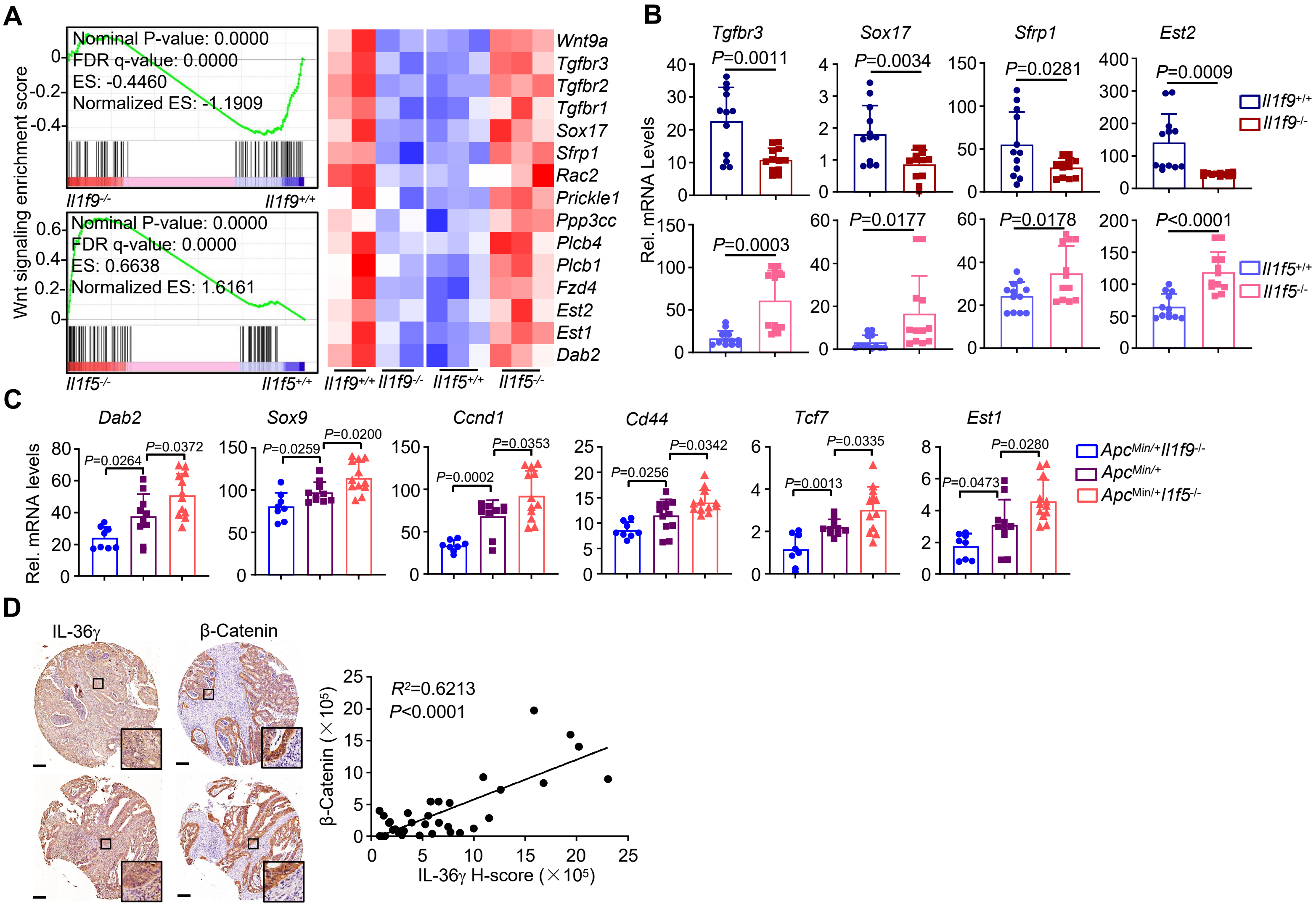

Supplementary Figure 5 Knockout of IL-36γ and IL-36Ra inhibits and promotes the expression of Wnt signaling during colon tumorigenesis, respectively.

(A) GSEA analysis (left) and heatmap (right) of the genes involved in Wnt signaling pathways from *Il1f9*$^{+/+}$ (n=2) and *Il1f9*$^{-/-}$ (n=2) mice that were given 2.5% DSS in drinking water for 5 d, followed by normal drinking water for another 2 d or *Il1f5*$^{+/+}$ (n=3) and *Il1f5*$^{-/-}$ (n=3) mice that were given 2% DSS in drinking water for 5 d, followed by normal drinking water for another 2 d.

(B) qRT-PCR analysis of the indicated genes in the inflamed colon tissues of *Il1f9*$^{+/+}$ (n=12) and *Il1f9*$^{-/-}$ (n=12) mice or *Il1f5*$^{+/+}$ (n=12) and *Il1f5*$^{-/-}$ (n=12) mice that were treated as in (A).

(C) qRT-PCR analysis of the indicated genes in the colon tumors of 5-month-old *Apc*$^{Min/+}$ (n=10), *Apc*$^{Min/+}$*Il1f9*$^{-/-}$ (n=8) and *Apc*$^{Min/+}$*Il1f5*$^{-/-}$ (n=12) mice.

(D) IHC staining (left) and Pearson correlation analysis (right) of IL-36γ and β-Catenin in human CRC biopsies (n=36).

ES, enrichment score; NES, non-enrichment score; FDR, false discovery rate; FWER, family-wise error rate. Graphs show mean ± SEM. (B, C). Two-tailed student's *t*-test in B, C. Scale bars represent 0.4 mm (D). Data are combined two independent experiments (B, C).

Supplementary Figure 6

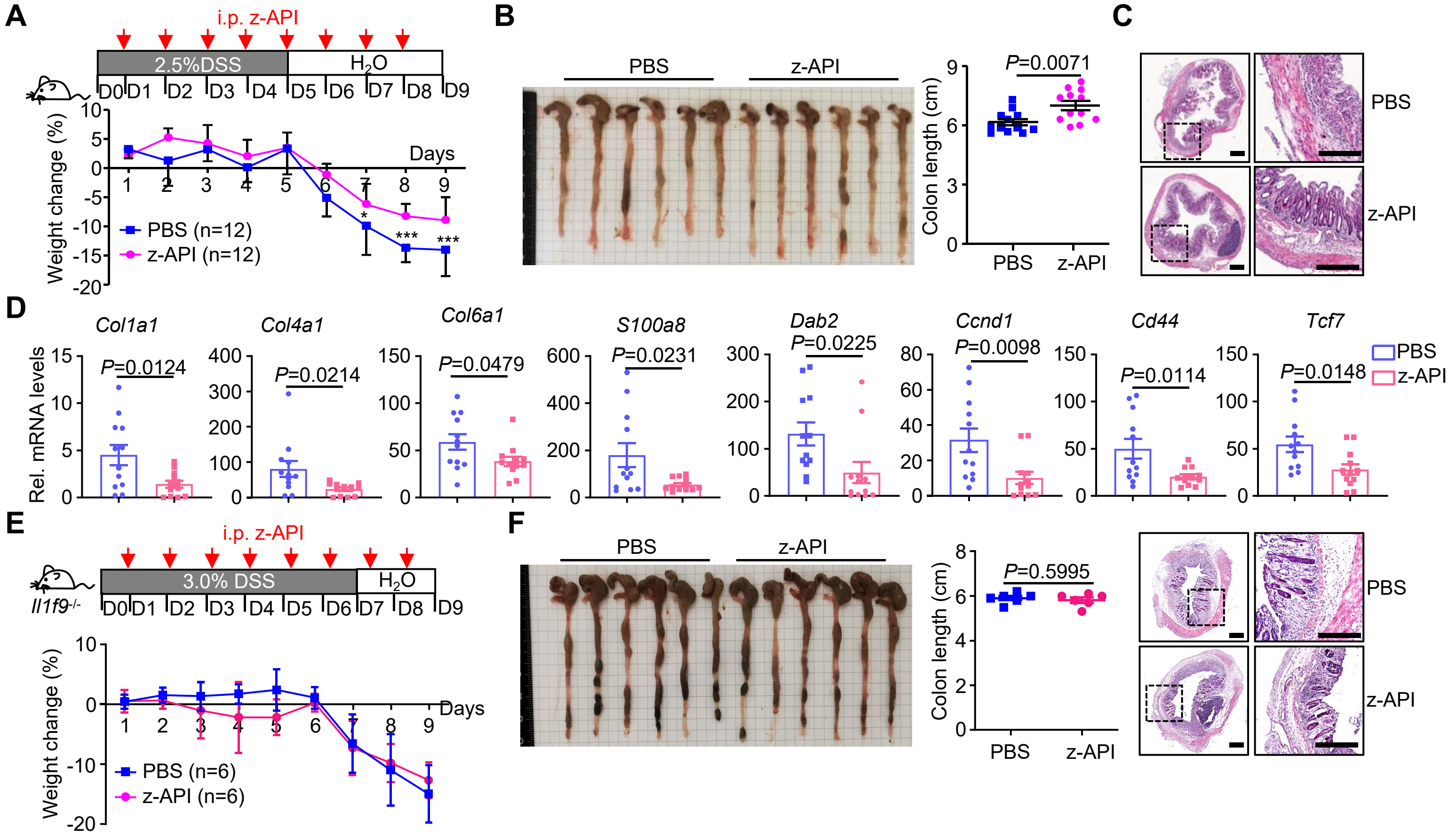

Supplementary Figure 6 Inhibition of IL-36γ maturation alleviates DSS-induced colitis.

(A) A scheme of DSS and z-API treatment (upper) and body weight change (lower) of wild-type mice that were fed with 2.5% DSS for 5 d followed by normal sterile water for 2 d and were intraperitoneally injected with PBS (n=12 mice) or z-API (100 μg) per mouse (n=12 mice) every day for seven successive days.

(B) Morphological change of representative colons (left) and colon lengths (right) of mice treated as in (A) (n=12 for PBS and z-API groups).

(C) Images of HE stained colon sections from the mice treated as in (A).

(D) qRT-PCR analysis of the indicated genes in the colon tissues of the mice treated as in (A) (n=12 for PBS and z-API groups).

(E) A scheme of DSS and z-API treatment (upper) and body weight change (lower) of *Il1f9*$^{-/-}$ mice that were fed with 3% DSS for 7 d followed by normal sterile water for 2 d and were intraperitoneally injected with PBS (n=6 mice) or z-API (100 μg) per mouse (n=6 mice) every day for seven successive days.

(F) Morphological change of representative colons (left) and colon lengths (right) of mice treated as in (E) (n=6 for PBS and z-API).

(G) Images of HE stained colon sections from mice treated as in (E).

Two-tailed student's *t*-test (A, B, D-F). Scale bars represent 0.4 mm (C, G). Graphs show mean ± SEM. (A, B, D-F). Data are combined two (A-D) or representative of two (E, F) independent experiments.

Supplementary Figure 7

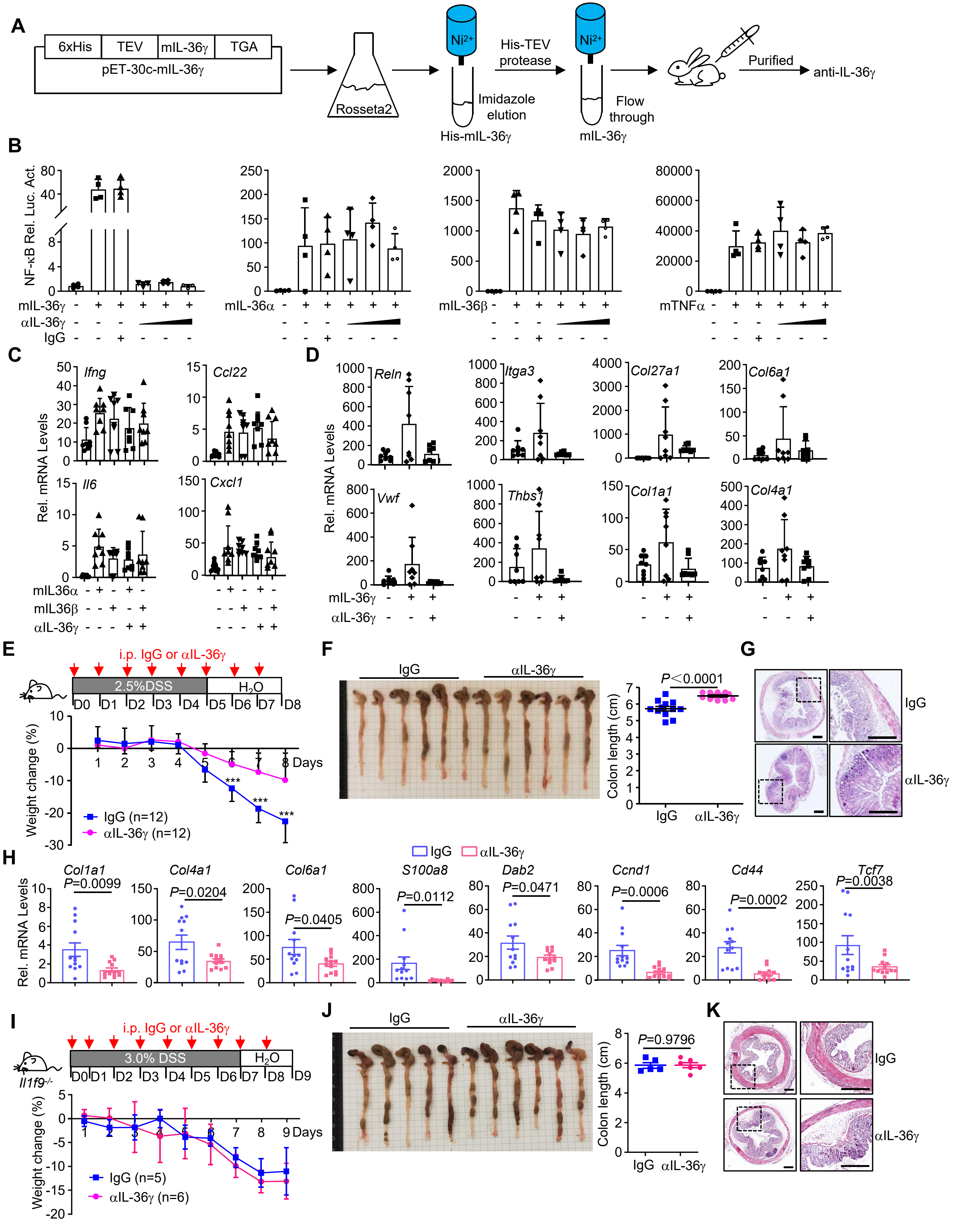

Supplementary Figure 7 The αIL-36γ specifically blocks mIL-36γ-triggered signaling.

(A) A scheme of mIL-36γ purification and immunization and the affinity purification of anti-IL-36γ.

(B) Luciferase assays of HEK293 cells that were transfected with mIL-36R for 24 h followed by stimulation with mIL-36γ, mIL-36α, mIL-36β (20 ng/ml), or mTNFα (5 ng/ml) together with control IgG or αIL-36γ (50 ng/ml) for 8 h.

(C-D) qRT-PCR analysis of the indicated genes of wild-type C57BL/6 colon organoids (n=8) stimulated with mIL-36α, mIL-36β or mIL-36γ (20 ng/ml) together withor without αIL-36γ (50 ng/ml) for 4 h.

(E) A scheme of treatment with αIL-36γ (100 μg) or control IgG (upper) (n=12 for IgG and αIL-36γ groups) and weight change (lower) of wild-type C57BL/6 mice that were given 2.5% DSS in drinking water for 5 d, followed by normal drinking water for another 3 d.

(F-G) Morphological change of representative colons (F, left) and colon lengths (F, right) and representative images of HE stained colon sections (G) from mice treated as in (E).

(H) qRT-PCR analysis of the indicated genes in the colon tissues of mice treated as in (E) (n=12 for IgG and αIL-36γ groups).

(I) A scheme of DSS-induced colitis with IgG or αIL-36γ treatment (upper) and body weight change (lower) of *Il1f9*$^{-/-}$ mice that were fed with 3% DSS for 7 d followed by normal sterile water for 2 d and were intraperitoneally injected with control IgG (n=5 mice) or αIL-36γ (100 μg) per mouse (n=6 mice) every day for nine successive days.

(J) Morphological change of representative colons (left) and colon lengths (right) of mice treated as in (I) (n=5 or 6 mice for IgG or αIL-36γ, respectively).

(K) Images of HE stained colon sections from mice treated as in (I).

***P<0.001 (E). Graphs show mean ± SEM. in (B-J). Two-tailed student's *t*-test (B-J). Scale bars represent 0.4 mm (G, K). Data are representative of two independent experiments (B-D, I-K) or combined two independent experiments (E-H).

Supplementary Figure 8

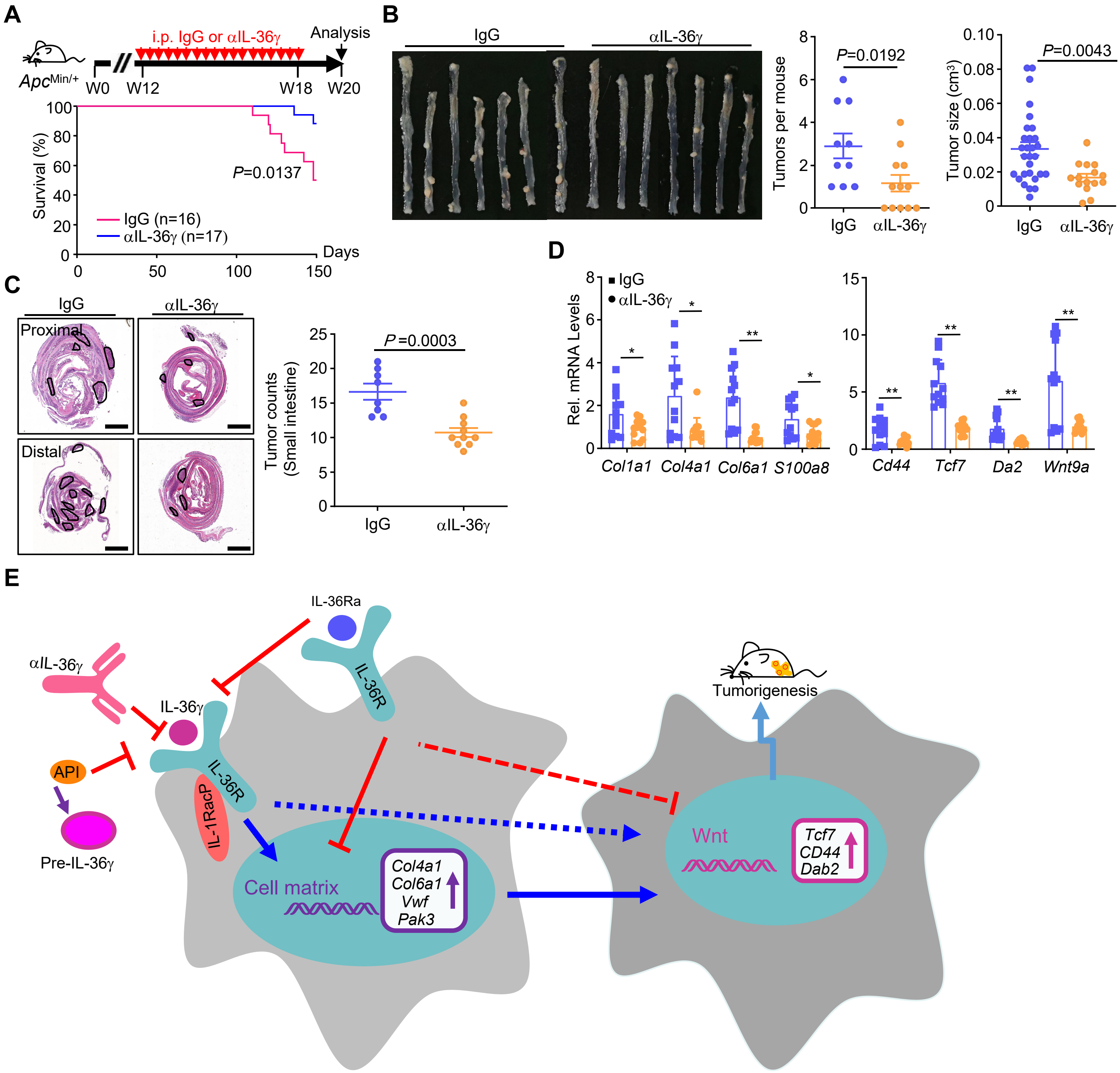

Supplementary Figure 8 Neutralization of IL-36γ inhibits tumorigenesis of *Apc*$^{Min/+}$ mice.

(A) A scheme of IgG or αIL-36γ treatment with *Apc*$^{Min/+}$ mice (upper). Survival (lower) of *Apc*$^{Min/+}$ mice (12-week-old) that were intraperitoneally injected with αIL-36γ (100 μg) per mouse (n=16 mice) or IgG (n=17 mice) every other day for 6 weeks followed by survival observation or rested for 2 weeks followed by analysis.

(B) Images (left), tumor counts (middle), tumor size (right) of colons from *Apc*$^{Min/+}$ mice treated as in (A) (n=10 or 12 for IgG or αIL-36γ, respectively).

(C) HE staining (C, left) and quantification analysis (C, right) of tumors in the small intestines of *Apc*$^{Min/+}$ mice treated as in (A) (n=8 for IgG and n=10 for αIL-36γ, respectively).

(D) qRT-PCR analysis of the indicated genes in the colon tumors of *Apc*$^{Min/+}$ mice treated as in (A) (n=12 for IgG or αIL-36γ, respectively).

(E) A model on IL-36γ- and IL-36Ra-mediated reciprocal regulation of colon cancer development. IL-36γ upregulates the expression of ECM and cell-matrix adhesion genes and synergizes Wnt signaling to promote tumorigenesis, which is mitigated by IL-36Ra. Therefore, targeting IL-36γ either by small molecules or by neutralizing antibodies effectively remodels ECM and cell-matrix interaction and inhibits colon cancer progression.

*$P < 0.05$; **$P < 0.01$ (log-rank analysis in A, two-tailed student's *t*-test in B-D). Graphs show mean ± SEM. in (B-D). Scale bars represent 1 mm (C). Data are combined two independent experiments (A-D).
