# Supplementary material for: IL‐36γ and IL‐36Ra Reciprocally Regulate Colon Inflammation and Tumorigenesis by Modulating the Cell–Matrix Adhesion Network and Wnt Signaling
Source: Adv Sci (Weinh). 2022 Feb 4;9(10):2103035. doi: 10.1002/advs.202103035 (PMC8981487; doi:10.1002/advs.202103035)

ADVANCED SCIENCE
Open Access

Supporting Information

for *Adv. Sci.*, DOI 10.1002/advs.202103035

IL-36γ and IL-36Ra Reciprocally Regulate Colon Inflammation and Tumorigenesis by Modulating the Cell–Matrix Adhesion Network and Wnt Signaling

Wei Yang, Hong-Peng Dong, Peng Wang, Zhi-Gao Xu, Jiahuan Xian, Jiachen Chen, Hai Wu, Yang Lou, Dandan Lin and Bo Zhong**

4d

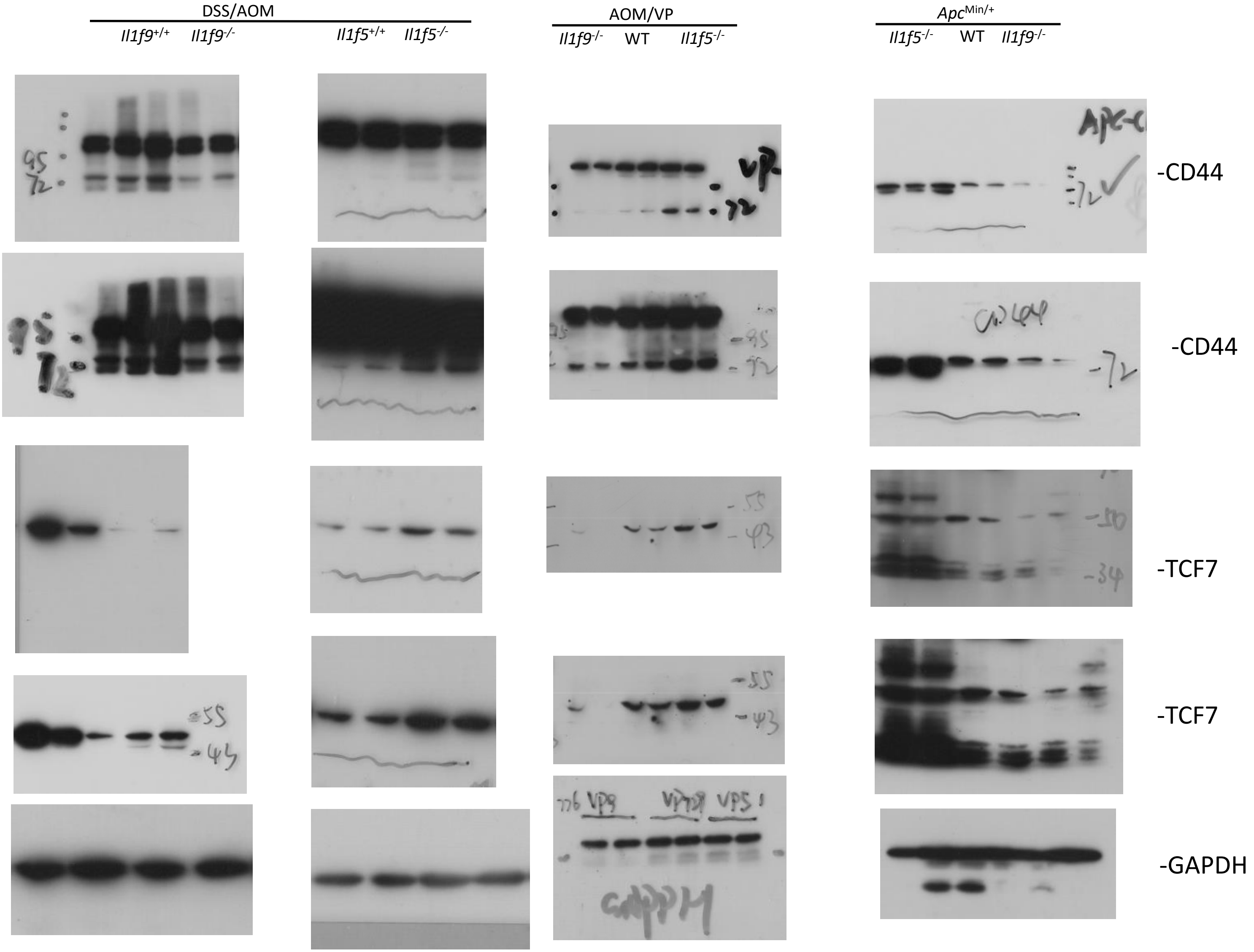

DSS/AOM
Il1f9+/+ Il1f9-/-
Il1f5+/+ Il1f5-/-
AOM/VP
Il1f9-/- WT Il1f5-/-
ApcMin/+
Il1f5-/- WT Il1f9-/-
-CD44
-CD44
-TCF7
-TCF7
-GAPDH

5h

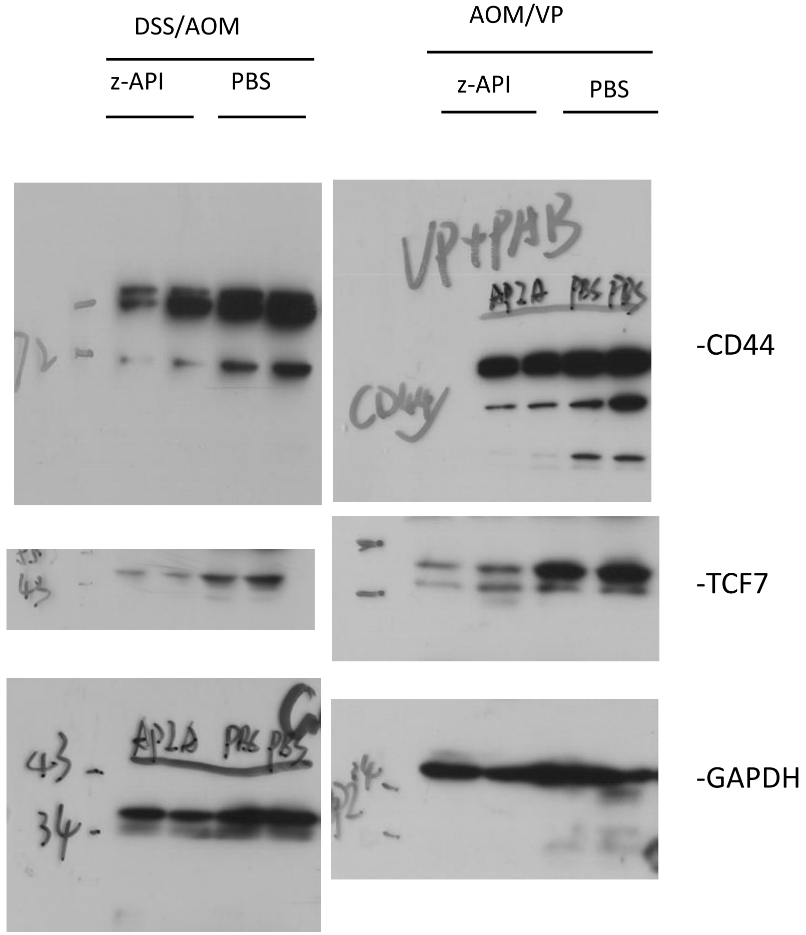

6g

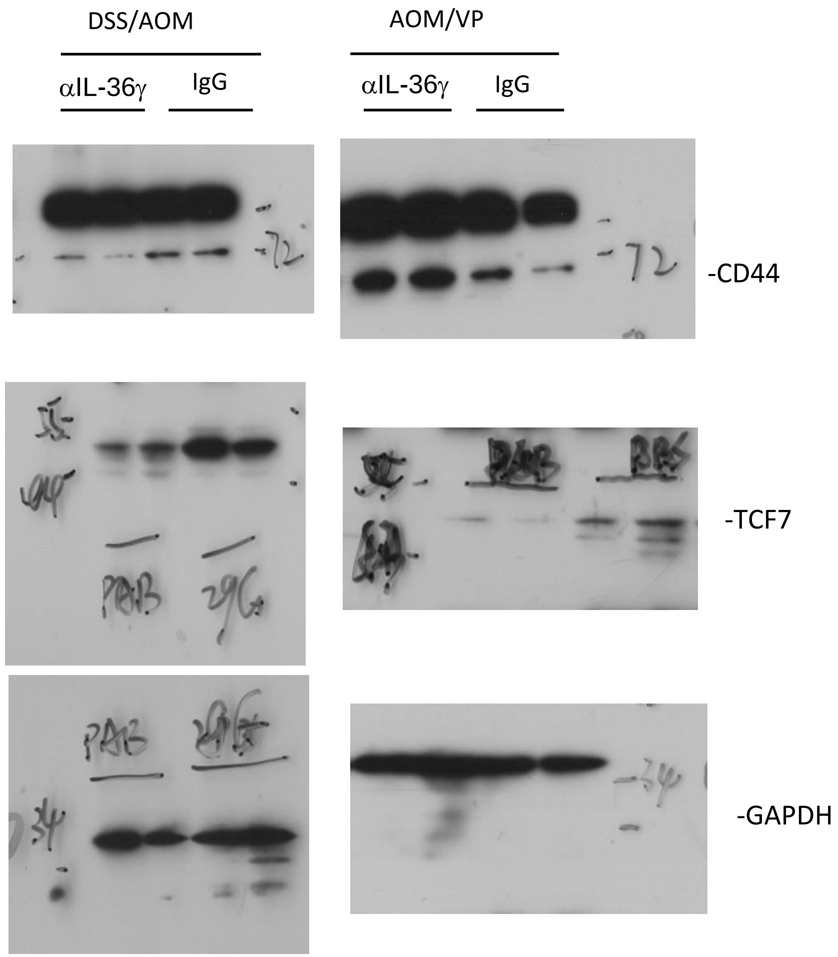

Supplement: Supplementary file 9 — Supporting Figure 1 [file ADVS-9-2103035-s008.pdf]
